# Blinatumomab-induced T cell activation at single cell transcriptome resolution

**DOI:** 10.1186/s12864-021-07435-2

**Published:** 2021-03-01

**Authors:** Yi Huo, Zhen Sheng, Daniel R. Lu, Daniel C. Ellwanger, Chi-Ming Li, Oliver Homann, Songli Wang, Hong Yin, Ruibao Ren

**Affiliations:** 1 Shanghai Institute of Hematology, State Key Laboratory for Medical Genomics, National Research Center for Translational Medicine, Collaborative Innovation Center of Hematology, RuiJin Hospital affiliated to Shanghai Jiao Tong University School of Medicine, Building 11, No. 197, Ruijin No.2 Rd, Shanghai, 200025 P.R. China; 2 Amgen Asia R&D Center, Amgen Biopharmaceutical R&D (Shanghai) Co., Ltd., 13F, Building 2, No. 4560, Jinke Rd, Shanghai, 201210 P.R. China; 3 Genome Analysis Unit, Amgen Research, Amgen Inc.,, South San Francisco, California, USA

**Keywords:** Bi-specific T-cell engager antibody, Acute B cell lymphoblastic leukemia, Blinatumomab, T cell activation, Single-cell RNA-Seq, TNFRSF4

## Abstract

**Background:**

Bi-specific T-cell engager (BiTE) antibody is a class of bispecific antibodies designed for cancer immunotherapy. Blinatumomab is the first approved BiTE to treat acute B cell lymphoblastic leukemia (B-ALL). It brings killer T and target B cells into close proximity, activating patient’s autologous T cells to kill malignant B cells via mechanisms such as cytolytic immune synapse formation and inflammatory cytokine production. However, the activated T-cell subtypes and the target cell-dependent T cell responses induced by blinatumomab, as well as the mechanisms of resistance to blinatumomab therapy are largely unknown.

**Results:**

In this study, we performed single-cell sequencing analysis to identify transcriptional changes in T cells following blinatumomab-induced T cell activation using single cells from both, a human cell line model and a patient-derived model of blinatumomab-mediated cytotoxicity. In total, the transcriptome of 17,920 single T cells from the cell line model and 2271 single T cells from patient samples were analyzed. We found that CD8+ effector memory T cells, CD4+ central memory T cells, naïve T cells, and regulatory T cells were activated after blinatumomab treatment. Here, blinatumomab-induced transcriptional changes reflected the functional immune activity of the blinatumomab-activated T cells, including the upregulation of pathways such as the immune system, glycolysis, IFNA signaling, gap junctions, and IFNG signaling. Co-stimulatory (TNFRSF4 and TNFRSF18) and co-inhibitory (LAG3) receptors were similarly upregulated in blinatumomab-activated T cells, indicating ligand-dependent T cell functions. Particularly, B-ALL cell expression of TNFSF4, which encodes the ligand of T cell co-stimulatory receptor TNFRSF4, was found positively correlated with the response to blinatumomab treatment. Furthermore, recombinant human TNFSF4 protein enhanced the cytotoxic activity of blinatumomab against B-ALL cells.

**Conclusion:**

These results reveal a target cell-dependent mechanism of T-cell activation by blinatumomab and suggest that TNFSF4 may be responsible for the resistant mechanism and a potential target for combination therapy with blinatumomab, to treat B-ALL or other B-cell malignancies.

**Supplementary Information:**

The online version contains supplementary material available at 10.1186/s12864-021-07435-2.

## Background

Over the past three decades, standard chemotherapy has improved the prognosis of adult patients with acute lymphoblastic leukemia (ALL). However, more than half of these patients are either refractory to therapies or relapse (r/r ALL) [1, 2]. Overall, patients with r/r ALL still have a poor prognosis after allogeneic hematopoietic stem-cell transplant [3–8]. Blinatumomab, a bispecific T cell engager antibody (BiTE) targeting both CD3 and CD19, has displayed clinical activities in patients with r/r B-ALL in different clinical trials [9–14]. In a recent phase-3 trial comparing blinatumomab to standard chemotherapy, the blinatumomab group (7.7 months) achieved a longer overall median duration of remission than the chemotherapy group (4.0 months). In addition, full hematologic recovery occurred significantly more frequently in the blinatumomab group than in the chemotherapy group [9].

Despite these encouraging results, not all patients with B-ALL respond to blinatumomab therapy based on evaluations of the response rate to blinatumomab in multiple studies [9, 12, 15–17]. For example, in a phase-2 study involving 189 adult patients with Philadelphia chromosome (Ph)-negative r/r B-ALL the overall response rate was 43%, similar to that observed in a multi-institutional phase-3 trial, where this response was 44%. It is currently unknown why T cells are able to kill tumor cells in some cases but remain unresponsive in others.

Blinatumomab connects T cells and target cells, forming immunologic synapses that potently trigger CD3-transduced signaling cascades in T cells [18, 19]. However, unlike typical T cell activation, blinatumomab-induced activation occurs independently of MHC I and additional T cell co-stimulatory factors, such as anti-CD28 antibody and interleukin-2. Notably, T cells cannot be activated by blinatumomab nor other BiTE antibodies in the absence of target cells [20]. In addition, BiTE antibody-mediated T cell functions are target cell-dependent [21].

Although blinatumomab’s mode of action of has been studied in different models [20, 22–25], the mechanism underlying target cell-dependent T cell response to blinatumomab remains largely uncharacterized. Previous studies have shown that PD-L1 was upregulated on leukemia blasts from a patient with resistance to blinatumomab treatment. Blinatumomab-mediated T cell functions were regulated by PD-L1 and CD80/CD86 on tumor cells, which, in turn, limit the cytolytic activity of blinatumomab [21, 26, 27]. Moreover, the effect of PD-L1 blockade on the enhancement of blinatumomab-mediated cytotoxicity strictly relies on the expression of PD-L1 [28]. However, there are still patients showing a poor response to blinatumomab therapy even in the presence of the immune checkpoints inhibitors PD-1 and CTLA4 [29]. These results suggest that a limited activity of these combination therapies in cases of blinatumomab resistance. Importantly, this necessitates the implementation of in-depth studies aimed to discover the key factors accounting for blinatumomab resistance.

The proliferation of both CD8+ and CD4+ T cells induced by blinatumomab or other BiTE antibodies has been previously detected by flow cytometry. Effector memory T (T_EM_) cells are the major subpopulation amongst these proliferating T cells, and the proportions of naïve T cells, central memory T (T_CM_) cells and CD45RA+ T_EM_ cells remain unchanged. Therefore, it has been hypothesized that T_EM_ cells account for most of the blinatumomab-mediated cytotoxicity [14, 24, 30, 31]. In addition, the priming and activation of naïve T cells strongly rely on signaling through CD28 and other co-stimulatory molecules [32], leading to the conclusion that naïve T cells will not be activated by blinatumomab in the absence of any costimulatory factors. Conversely, other studies have shown that the cytotoxicity from BiTE antibodies is mediated by various T cell populations, including regulatory T cells (Tregs), which inhibit T cell-engaged specific lysis during blinatumomab treatment of B-ALL [33, 34]. Accordingly, in-depth analysis on T-cell populations is required in order to comprehensively understand their dynamic changes upon blinatumomab treatment.

Recently, single-cell RNA-seq (scRNA-seq) has been widely used in the analysis of T cell subpopulations [35–37]. The rapid development of scRNA-seq analysis allows us to dissect complex cell populations and explore the heterogeneity of T cell responses to blinatumomab treatment at a higher resolution. In this study, scRNA-seq analysis was used to investigate the responses of different T cell populations and the mechanism of target cell-dependent T cell responses induced by blinatumomab.

## Results

### Single-cell transcriptional profiling of a blinatumomab-mediated cytotoxicity model

**Fig. 1:**
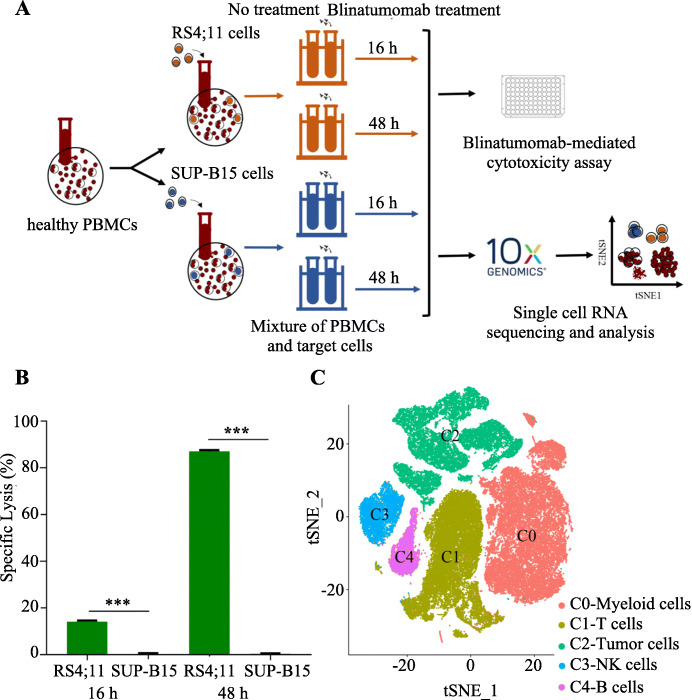
Blinatumomab induced B-ALL cytotoxicity model for single cell profiling. **a** Schematic of study of blinatumomab-mediated cytotoxicity in a cell line model. **b** Specific lysis of target cells after treatment with 0.1 ng/mL blinatumomab for 16 and 48 h. The experiment was conducted in three independent replicates. *P < 0.05; **P < 0.01; ***P < 0.001; two-sided paired Student’s t-test. **c** A T-distributed stochastic neighbor embedding (t-SNE) projection of all single cells from a cell line model with 5 main clusters in different colors. The identity of each cluster was determined based on its signature genes

In order to assess the effect of blinatumomab on T cell responses ex vivo, target cells from RS4;11 and SUP-B15 B-ALL cell lines were co-cultured with healthy PBMCs and 0.1 ng/mL blinatumomab for 16 or 48 h, which was followed by a blinatumomab-mediated cytotoxicity assay and scRNA-seq (Fig. 1a). As shown in Fig. 1b, the percentage specific lysis of RS4;11 cells were significantly higher than that of SUP-B15 cells upon blinatumomab treatment., suggesting the difference in blinatumomab sensitivity between these two cell lines. The dose-dependent specific lysis induced by blinatumomab in RS4;11 and SUP-B15 cells are shown in Additional file 1, Fig. S1A-B.

For single-cell transcriptome analysis, a total of 64,613 cells met the data quality requirements and were subsequently normalized, batch corrected, and analyzed (sample information and detailed cell number for each sample are listed in Additional file 1, Table S1). Data from different conditions mixed well, implying proper data integration (Additional file 1, Fig. S1C). By applying unsupervised clustering in the principal component space of this dataset, we identified five cell clusters (Fig. 1c). Cluster C1 was composed of T cells exhibiting a highly specific expression of the T cell markers *CD3D* and *CD3E*. Clusters C0, C2, C3 and C4 were defined as myeloid cells, tumor cells, NK cells and B cells, respectively, based on their expression of well-known markers, such as CD14 / S100A9 / LYZ, CD79A, NKG7 / FCGR3A and MS4A1 / CD79A (Additional file 1, Fig. S1D).

### Unsupervised clustering and identification of blinatumomab-responsive T cell populations

**Fig. 2:**
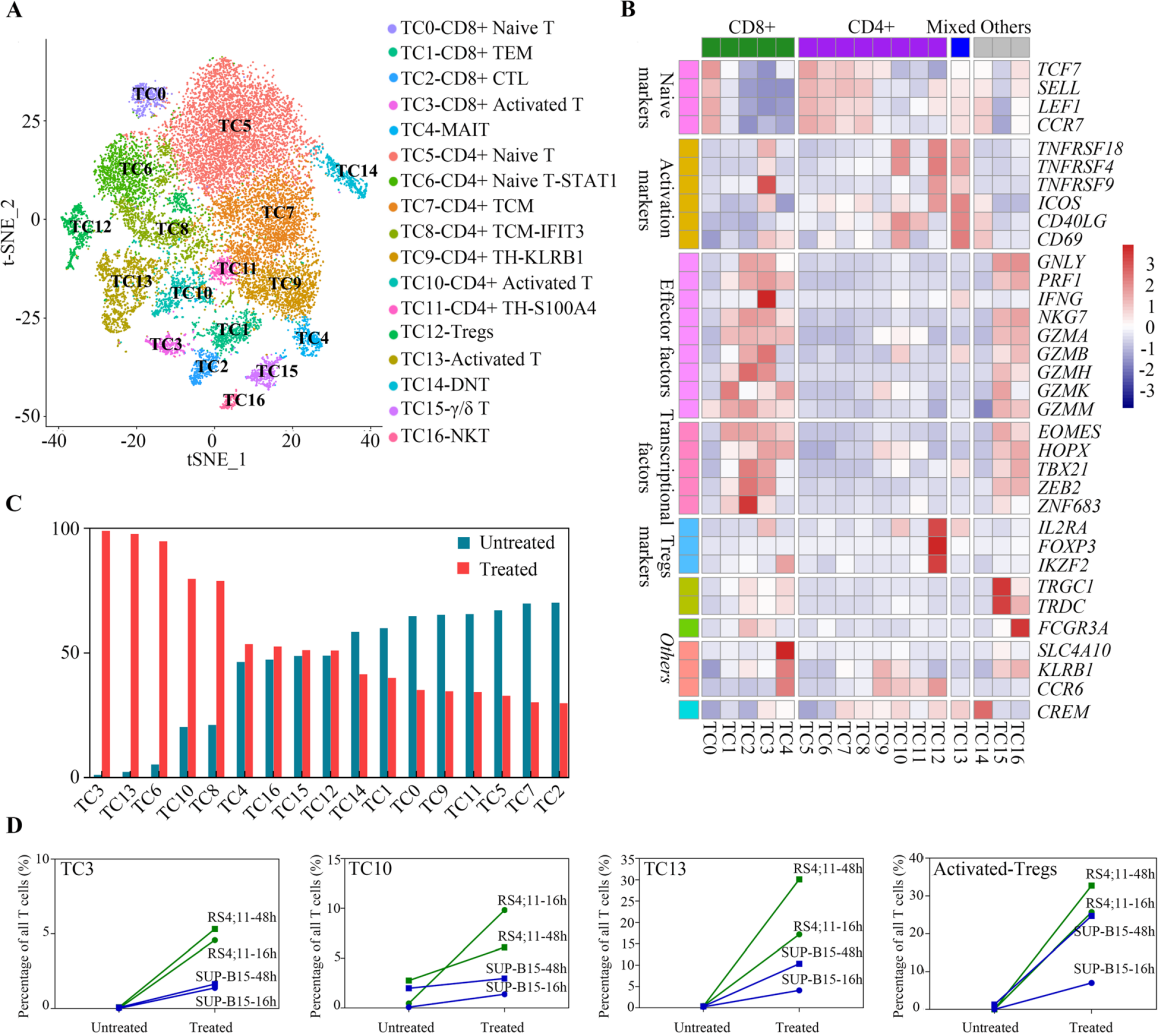
Characterization of T cell subtypes in B-ALL cytotoxicity model. **a** The t-SNE projection of all T cells identified in Fig. 1b 17 subclusters are highlighted in different colors. The identity of each cluster was determined based on its signature genes. **b** The Z-score normalized mean expression of selected genes in each T cell subcluster. **c** The proportion of each cluster in the untreated (RU-16 h, RU-48 h, SU-16 h, and SU-48 h) and blinatumomab-treated (RT-16 h, RT-48 h, ST-16 h, and ST-48 h) groups. The clusters were placed in descending order based on the proportion of each cluster in the blinatumomab-treated group. **d** The percentage of activated TC3-CD8+, activated TC10-CD4+, and activated TC13-activated T cells among the total T cells and the percentage of activated regulatory T cells (Tregs) among the total Tregs in each sample. RS4;11-16 h represents RU-16 h and RT-16 h. RS4;11-48 h represents RU-48 h and ST-48 h. SUP-B15-48 h represents SU-48 h and ST-48 h. SUP-B15-16 h represents SU-16 h and ST-16 h

To characterize the intrinsic response of T cells to blinatumomab treatment, we further assessed the 17,920 T cells comprising cluster C1 from four untreated samples (RU-16 h: untreated RS4;11 cells at 16 h, RU-48 h: untreated RS4;11 cells at 48 h, SU-16 h: untreated SUP-B15 cells at 16 h, and SU-48 h: untreated SUP-B15 cells at 48 h) and four blinatumomab-treated samples (RT-16 h: treated RS4;11 cells at 16 h, RT-48 h: treated RS4;11 cells at 48 h, ST-16 h: treated SUP-B15 cells at 16 h, and ST-48 h: treated SUP-B15 cells at 48 h). One cluster mainly contained cell doublets (80 cells, cluster 17 in Additional file 1, Fig. S2A) and, therefore, was not included in downstream analysis due to the significantly larger number of total detected genes and UMIs per cell compared to other clusters (Additional file 2, Fig. S2B-C). Finally, 17 sub-clusters with corresponding signature genes were identified in an unbiased manner (Fig. 2a, Additional file 2, Table S2). Based on the distribution of *CD4* and *CD8* expression (Additional file 1, Fig. S3A) and *CD4*/*CD8* ratios (Additional file 1, Fig. S3B), we identified five CD8+ T cell clusters (TC0-TC4), eight CD4+ T cell clusters (TC5-TC12), and one CD4+/CD8+ mixed T cell cluster (TC13). The cell type within each cluster was assessed based on the expression of several known functional markers (Fig. 2b). The five CD8+ T cell clusters were defined as naïve T cells (TC0-CD8+ Naive T), T_EM_ cells (TC1-CD8+ TEM), cytotoxic T lymphocytes (TC2-CD8+ CTL), activated T cells (TC3-CD8+ Activated T), and mucosa-associated invariant T cells (TC4-MAIT). Similarly, CD4+ T cell clusters were defined as naïve T cells (TC5-CD4+ Naïve T and TC6-CD4+ Naïve T-STAT1), T_CM_ cells (TC7-CD4+ TCM and TC8-CD4+ TCM-IFIT3), activated T cells (TC10-CD4+ Activated T), and Tregs (TC12-Tregs). The CD4+/CD8+ mixed cluster was also defined as activated T cells (TC13-Activated T). The remaining clusters were annotated as double-negative T cells (TC14-DNT), gamma/delta T cells (TC15-gamma/delta T), and natural killer T cells (TC16-NKT). Additional details on cell type identification are described in supplementary text (Additional file 3).

Interestingly, the TC12-Tregs cluster expressed the activation markers *TNFRSF4*, *TNFRSF18* and *IL2RA* after blinatumomab treatment (Additional file 1, Fig. S4A). In order to further characterize the activated Treg cluster, unsupervised clustering was performed on all Tregs. A total of three distinct Treg clusters were identified without bias (Additional file 1, Fig. S4B) and defined as Resting-Tregs, IFN-Tregs and Activated-Tregs based on their distinct signature genes (Additional file 1, Fig. S4C).

The proportion of each T cell cluster (Fig. 2c) and Treg cluster (Additional file 1, Fig. S4D) in the combined untreated and blinatumomab-treated groups were compared to reveal T cell population changes. The TC6-CD4+ Naïve T-STAT1, TC8-CD4+ TCM-IFIT3 and IFN-Tregs clusters were highly enriched after blinatumomab treatment (Fig. 2c, Additional file 1, Fig. S4D), implying a T cell state transition induced by blinatumomab. Moreover, the clusters composed of activated T cells (TC3, TC10, TC13 and Activated-Tregs) were predominantly enriched after blinatumomab treatment (Fig. 2c, Additional file 1, Fig. S4D). The percentage of activated T cell clusters (Fig. 2d), as well as other blinatumomab-responsive clusters (Additional file 1, Fig. S5), were found to be higher in the RS4;11 group than in the SUP-B15 group after blinatumomab treatment for 16 and 48 h. This observation is in accordance with the differential blinatumomab-induced specific lysis (Fig. 1b). The result shown in Fig. 2c was further dissected by cell lines and time points (Additional file 1, Fig. S6). Our results show that the T cell changes in individual cell lines and at different time points are in agreement with the observations of the combined one. The blinatumomab responsive T cell clusters (TC3, TC10 and TC13) consistently expanded after blinatumomab treatment in both cell lines, while the magnitude of the expansions in the RS4;11 group were larger than those in SUP-B15 group at both 16 and 48 h. No significant differences were found between time points within specific cell line (RS4;11, *p*-value = 0.24, paired T test; SUP-B15, p-value = 0.08, paired T test). Overall, these results indicate that blinatumomab-responsive clusters play functional roles in blinatumomab-mediated cytotoxicity.

### Revealing blinatumomab induced T cell state transition

**Fig. 3:**
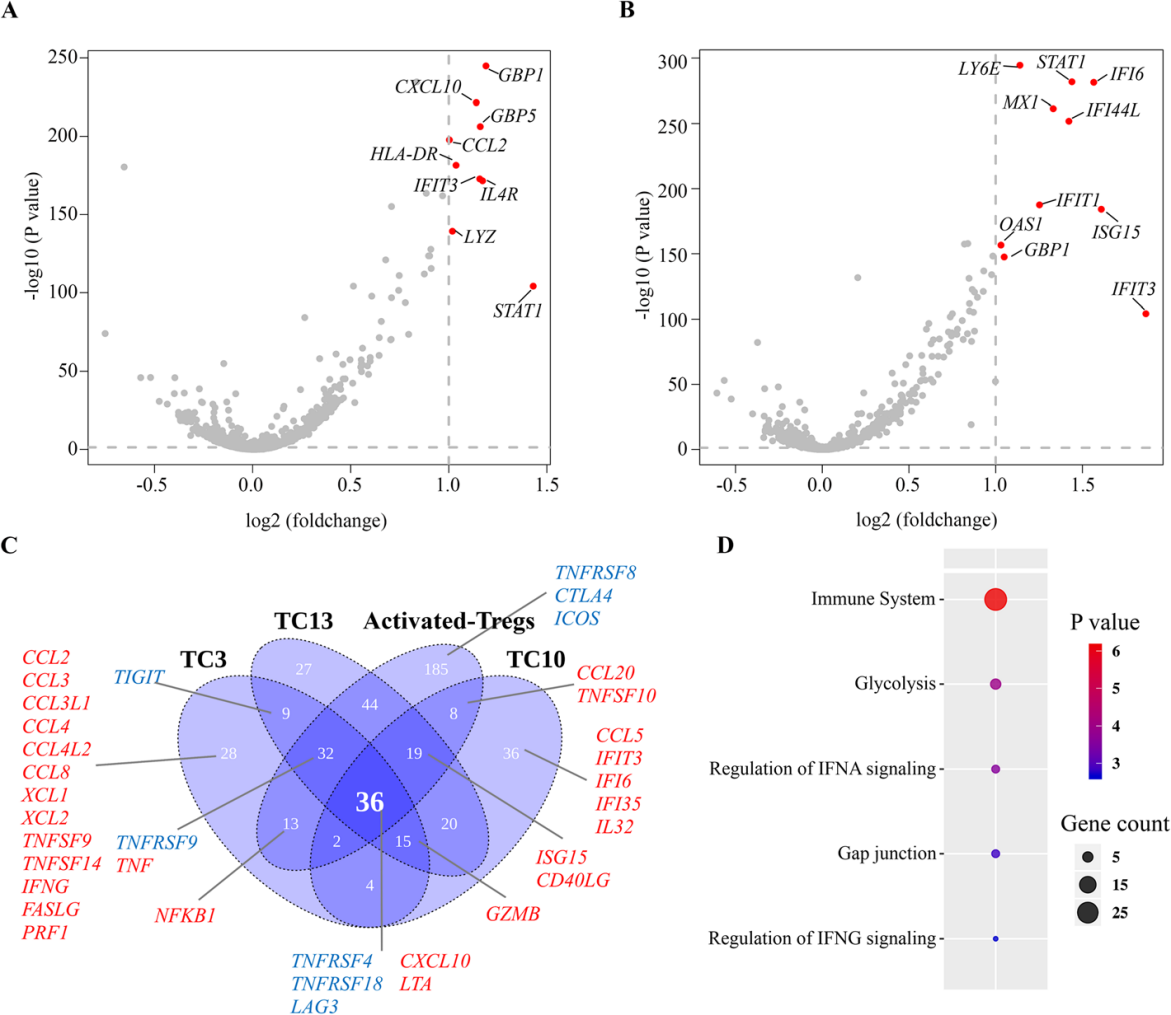
Blinatumomab-induced transcriptional changes and pathway analysis in T cell subclusters. **a**-**b** Volcano plot showing differentially expressed genes between **a** clusters TC5-CD4+ Naïve T and TC6-CD4+ Naïve T-STAT1, **b** clusters TC7-CD4+ TCM and TC8-CD4+ TCM-IFIT3. Genes with a *P* value < 0.05 and fold change > 2 are highlighted in red. **c** Venn plot showing the numbers of genes expressed differentially between the blinatumomab-activated and original clusters. Genes encoding cytokines and chemokines are labeled in red. Genes encoding co-signaling receptors are labeled in blue. TC3 represents the comparison between TC3-CD8+ Activated T and TC2-CD8+ TEM. TC13 represents the comparison between TC13-Activated T, TC0-CD4+ Naïve T, and TC5-CD4+ Naïve T. Activated-Tregs represents the comparison between Activated-Tregs and Resting-Tregs. TC10 represents the comparison between TC10-CD4+ Activated T and TC7-CD4+ TCM. **d** Pathway enrichment analysis revealed 36 differentially expressed genes listed in the reactome database (https://reactome.org/*).* The size of the circles is proportional to the Gene Count in the corresponding category. The color of each circle corresponds to the P value. Pathways are ranked according to their P value

In order to characterize the T cell state transition induced by blinatumomab, the three highly enriched TC6-CD4+ Naïve T-STAT1, TC8-CD4 + TCM-IFIT3 and IFN-Tregs clusters were compared with their respective original clusters, which are in their naïve or resting state. As shown in Fig. 3a, the comparison between TC6-CD4+ Naïve T-STAT1 with TC5-CD4+ Naïve T revealed that the TC6 cluster expressed higher levels of interferon (IFN)-induced genes, including *STAT1*, *GBP1*, *GBP5*, *IFIT3*, *CCL2*, *CXCL10* and *IL4R* [38–42]. Compared to the TC7-CD4+ TCM cluster, the TC8-CD4 + TCM-IFIT3 cluster also showed higher expression levels of genes associated with IFN responses, including *IFIT3*, *GBP1*, *IFIT1*, *IFI6*, *STAT1*, *IFI44L* and *MX1* (Fig. 3b) [43–48]. Similarly, IFN-Tregs cells exhibited higher transcript levels of IFN-responsive genes (including *IFIT3*, *IFIT6*, *ISG15*, *STAT1*, *EPSTI1* and *MX1*) than Resting-Tregs cells (Additional file 1, Fig. S4C). These results suggest that blinatumomab induces an IFN-responsive state transition associated with cytotoxicity.

We performed de novo alignment of T cells from the CD8+ and CD4+ T cell clusters along a pseudo-temporal axis representing the continuum of blinatumomab-induced activation. CD8+ cells formed a trajectory from the TC0-CD8+ Naïve T cluster, followed by clusters TC1-CD8+ TEM and TC2-CD8+ CTL towards cluster TC4-CD8+ Activated T (Additional file 1, Fig. S7A). Similarly, the TC10-CD4+ Activated T cluster was chronologically ordered at the terminal end of the CD4+ activation trajectory (Additional file 1, Fig. S7B). Genes associated with activation were mapped onto the activation trajectory, confirming an increased expression of the genes *IL2RA*, *CD69*, *TNFRSF18* and *TNFRSF4* at the end of the trajectories (Additional file 1, Fig. S7C-D). Both trajectories suggest that the activated CD8+ and CD4+ T cells did not originate from naïve T cells, but rather from CD8+ T_EM_ and CD4+ T_CM_ cells, respectively. By contrast, cells from the TC13-Activated T cluster expressed high levels of naïve marker genes (Fig. 2b), leading to the conclusion that cells from this CD8+/CD4+ mixed activated T cell cluster arose from naïve T cells (TC0 and TC5).

### Identification of transcriptional changes in blinatumomab activated T cells

In order to reveal the transcriptional changes during blinatumomab-induced T cell activation, the transcriptional profiles of cells from blinatumomab-activated clusters (TC3, TC10, TC13 and Activated-Tregs) were compared with untreated cells from their respective original clusters (TC1&TC7, TC0, TC5, and Resting-Tregs). The differentially expressed genes (DEGs) were identified and listed in Additional file 4, Table S3.

Thirty-six DEGs (*P* value < 0.05, fold change > 1.5) were common across all four blinatumomab-activated T cell clusters, although several unique DEGs were found in each of the activated T cell clusters (Fig. 3c, Additional file 4, Table S3). The majority of the common DEGs were enriched in pathways corresponding to immune system-related processes (Fig. 3d). Other genes were enriched in glycolysis, the regulation of IFNA signaling, gap junction, and the regulation of IFNG signaling pathways (Fig. 3d). Glycolysis enables the rapid proliferation and the effector function of activated T cells [49, 50], while gap junctions accumulate at immunological synapses, contributing to T cell activation [51]. The upregulation of glycolysis and gap junction pathways, as well as the enrichment of immune system-related processes, demonstrated that blinatumomab-activated T cells had a higher functional immune activity than their original cells, thus accounting for blinatumomab-mediated cytotoxicity.

The production of distinct cytolytic factors and cytokines was found in these activated T cell populations, reflecting their different functions (Fig. 3c). We found that *GZMB*, which encodes the main component in cytolytic granules, was upregulated in TC3-CD8+ Activated T, TC10-CD4+ Activated T and TC13-Activated T clusters, reflecting their cytolytic capability following blinatumomab activation. TC3 specifically expressed chemokines important for recruiting immune cells to the site of cytotoxicity (*CCL2*, *CCL3*, *CCL3L1*, *CCL4*, *CCL4L2*, *CCL8*, *XCL1* and *XCL2*) [52, 53]. TC3 was also enriched with ligands, including *TNFSF9* and *TNFSF14*, which are essential for signal transduction and the maintenance of T cell functions () [54], and cytotoxic factors (*IFNG*, *FASLG,* and *PRF1*) [55], indicating that blinatumomab-activated CD8+ T_EM_ cells have a stronger cytolytic ability than other activated T cells. Cytokines induced by interferons were also upregulated, including *CXCL10*, *ISG15*, *IFIT3*, *IFI35,* and *IFI6*, confirming the activation of IFNA and IFNG regulation signaling pathways in the blinatumomab-activated clusters.

Distinct co-signaling receptors were induced by blinatumomab in four activated T cell populations (Fig. 3c). Specifically, *TNFRSF9* was only upregulated in TC3; *TIGIT* was upregulated in both TC3 and TC13-Activated T compared with their respective original clusters; and *TNFRSF8*, *CTLA4* and *ICOS*, which regulate the immunosuppressive function of Tregs [56–59], were specifically upregulated in Activated-Tregs. Importantly, both co-stimulatory receptors *TNFRSF4* and *TNFRSF18*, and the co-inhibitory receptor *LAG3*, were upregulated in all blinatumomab-activated T cell populations (Fig. 3c), implying that *TNFRSF4*, *TNFRSF18* and *LAG3* may constitute potential targets for modulating blinatumomab-induced T cell responses.

### Identification of blinatumomab-activated T cell clusters in B-ALL patient-derived cytotoxicity model

**Fig. 4:**
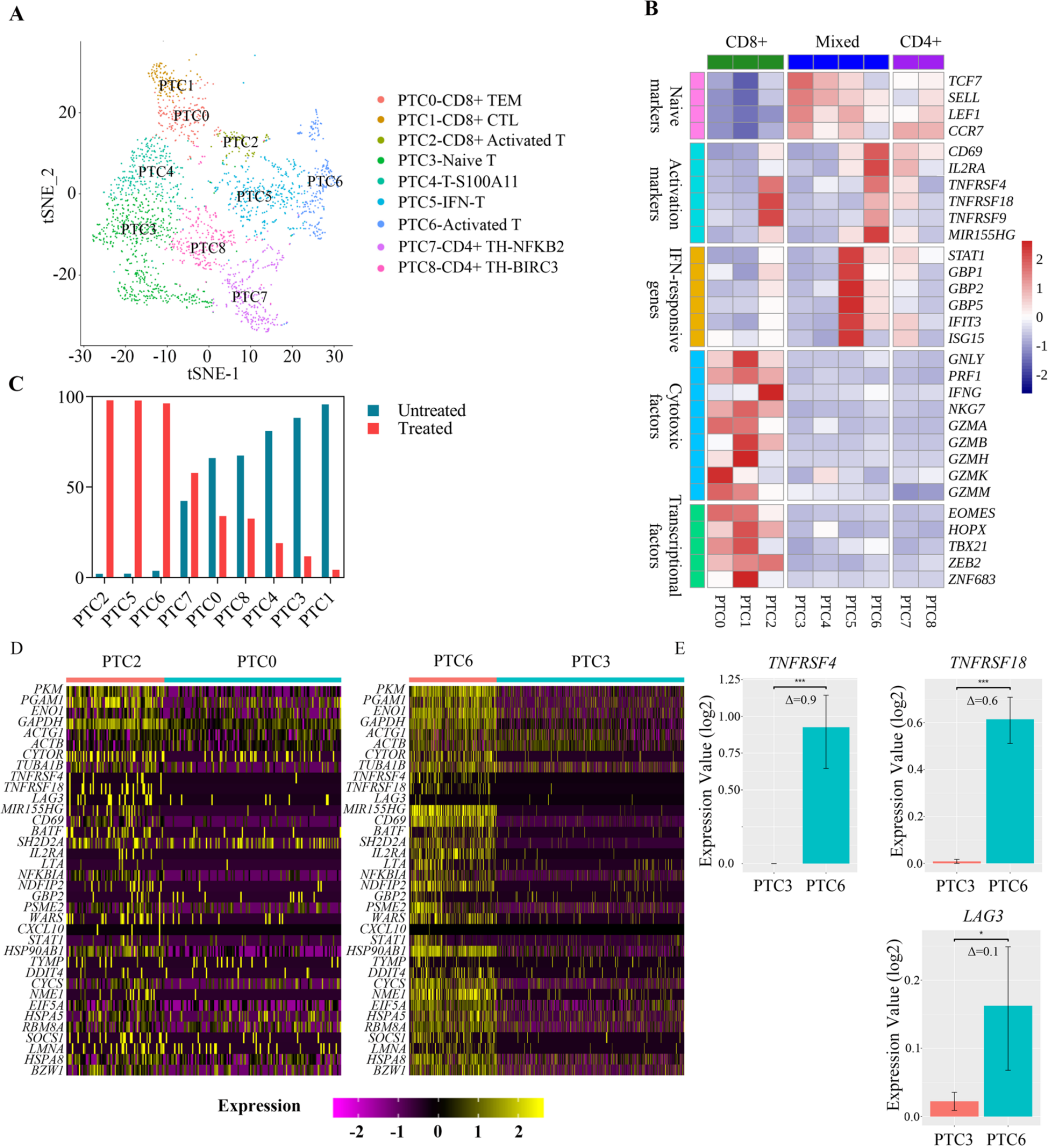
Characterization of T cell subtypes in patient derived B-ALL cytotoxicity model. **a** A t-SNE projection of all T cells in the patient derived model with 9 subclusters. The identity of each cluster was determined based on its signature genes. **b** Z-score normalized mean expression of signature genes in each cluster. **c** The proportion of each cluster in untreated (205BM-B0, 207BM-B0, and 207 L-B0) vs blinatumomab-treated (205BM-B50, 207BM-B50, and 207 L-B50) samples. **d** Heatmap of the 36 common genes identified in cell line models, differentially expressed between PTC2-CD8+ Activated and PTC0-CD8+ TEM, PTC6-Activated T and PTC3-Naïve T single cells. **e** Bar plot of the expression of *TNFRSF4*, *TNFRSF18* and *LAG3* in clusters PTC3-Naïve T and PTC6-Activated T. The y-axis shows the log2 value of the expression value. Δ represents the log2 value of the fold change

In order to validate the T cell responses in the cell line model in a more heterogeneous system, we analyzed a total of 2271 T cells from 13,240 sequenced single B-ALL PBMCs and BMMCs from two different donors (Additional file 1, Table S1, Fig. S8A-B), and nine T cell-clusters were identified with their signature genes (Fig. 4a, Additional file 5, Table S4). According to the expression of *CD4*, *CD8A, CD8B*, (Additional file 1, Fig. S8C) and other known functional markers (Fig. 4b) in each cluster, we defined three CD8+ T cell clusters as CD8+ T_EM_ cells (PTC0), cytotoxic T lymphocytes (PTC1), and CD8+ activated T cells (PTC2). PTC3, PTC5 and PTC6 were composed of naïve T cells, T cells with IFN response, and activated T cells, respectively. We calculated the numbers of shared signature genes of clusters from both cell line model and patient samples in order to compare the similarities of the relevant clusters from these two models (Additional file 1, Fig. S8D). The top 20 signature genes of activated clusters PTC2 (shared genes *N* = 9) and PTC6 (shared genes *N* = 10) were mostly similar to the corresponding clusters TC3-CD8+ Activated T and TC13-Activated T from the cell line models, respectively (Additional file 1, Fig. S8D). Furthermore, the proportion of PTC2, PTC6 and PTC5 increased after blinatumomab treatment (Fig. 4c). While both PTC2 and PTC6 showed population expansion after treatment, a different response of T cells to blinatumomab was found between patients #205 and #207 (Additional file 1, Fig. S9). This presumably occurred due to the heterogeneous T cell population and target malignant cells in those two patients. These results not only suggest that the T cell type composition, but also the transition to an IFN-responsive state and the T cell activation processes were comparable between the patient-derived model and the cell line model. In addition, the activated T cell clusters PTC2 and PTC6 also exhibit a high expression of the 36 common DEGs identified in the cell line model (Fig. 4d). This further confirmed the previously identified transcriptional changes in blinatumomab-activated T cells.

The two activated T cell clusters PTC2 and PTC6 both expressed higher levels of *TNFRSF4* than their untreated counter parts, PTC0 and PTC3 (Fig. 4e, Additional file 1, Fig. S8E). Additionally, the fold change of *TNFRSF4* (Δ = 0.9) in the PTC6 cluster was significantly higher than that of *TNFRSF18 (*Δ = *0.6)* or *LAG3* (Δ = 0.1) (Fig. 4c and Additional file 6, Table S5). This evident change in the amount of *TNFRSF4* underlines its functional roles in the modulation of blinatumomab-induced T cell activation.

### The effect of TNFRSF4 signaling on blinatumomab-induced cytotoxicity

**Fig. 5:**
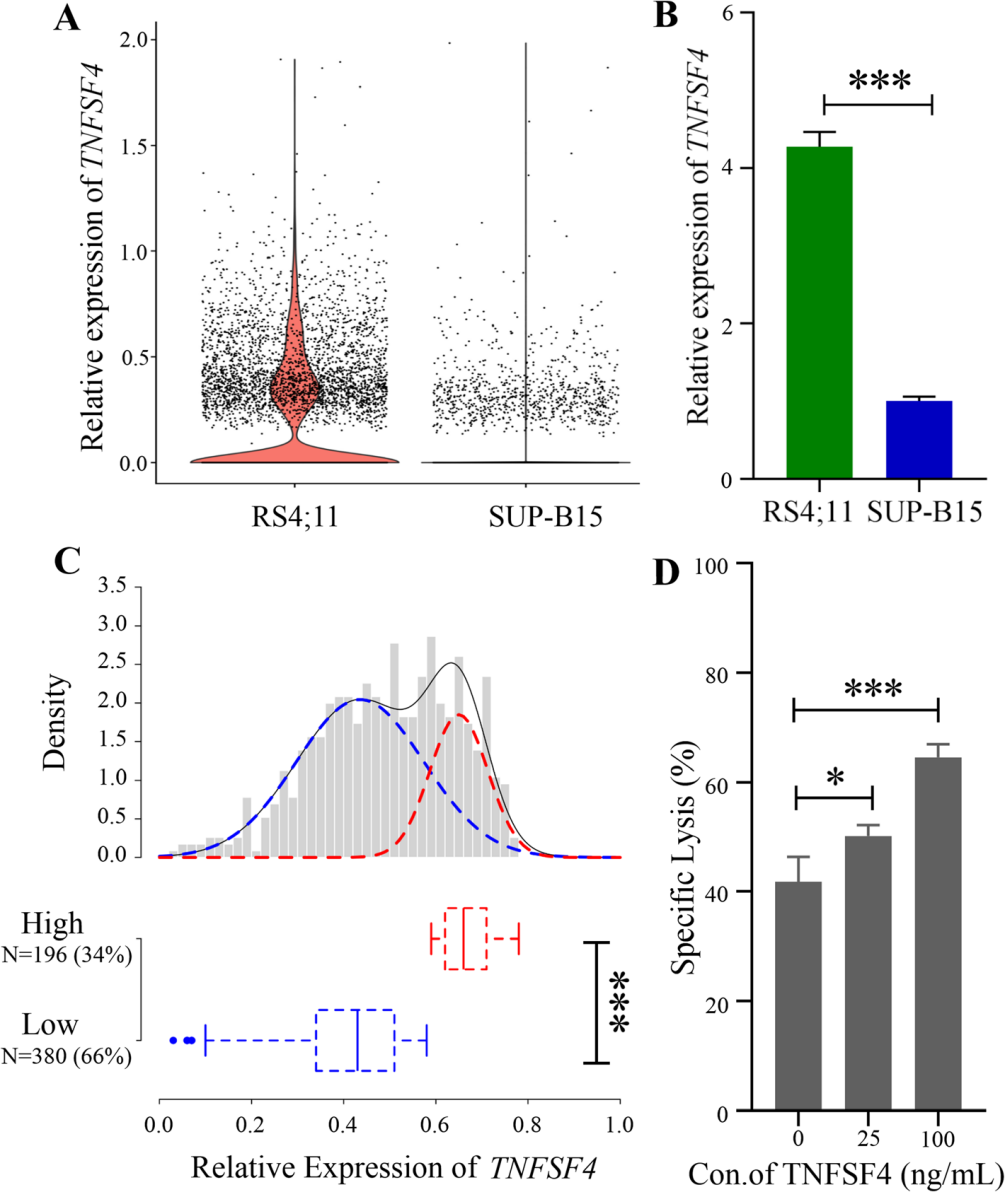
Differential expression of TNFSF4 in B-ALLs and its effect on blinatumomab mediated cytotoxicity. **a** Violin plot of relative TNFSF4 expression in RS4;11 and SUP-B15 cells based on scRNA-Seq data. **b** Relative expression of TNFSF4 in RS4;11 and SUP-B15 cells as measured by qPCR. GAPDH was the input control. The experiment was conducted in three independent replicates. *P < 0.05; **P < 0.01; ***P < 0.001; Student’s t-test. **c** The combined density distribution curve of the relative expression of TNFSF4 (probe set id: 207426_s_at) in 576 patients with B-ALL from Microarray Innovations in LEukemia study (GEO accession: GSE13204) using of 2-component Gaussian mixture model. The black solid curve represents 2-component Gaussian mixture model fitting. Dashed curves represents the 2-component distribution in the mixture model (blue: low expression, red: high expression). The boxplot shows the expression distribution of TNFSF4 in the two groups. *P < 0.05; **P < 0.01; ***P < 0.001; Student’s t-test. **d** Specific lysis of SUP-B15 cells after treatment with 0.1 ng/mL blinatumomab and recombinant human TNFSF4 proteins. The experiment was conducted in three independent replicates. *P < 0.05; **P < 0.01; ***P < 0.001; Student’s t-test

TNFSF4, which is the only known TNFRSF4 ligand, is constitutively expressed on antigen-presenting cells and transduces co-stimulatory signaling [60]. In order to corroborate the observed expression of *TNFSF4* on B-ALL tumor cells, we examined the expression of the gene in both B-ALL cell lines. The sensitive target cells, RS4;11, showed higher expression levels of *TNFSF4* than the less sensitive target cells, SUP-B15. These results were confirmed by q-PCR (Fig. 5a-b). The changes in the *TNFSF4* mRNA levels were also analyzed in the SUP-B15 and RS4;11 groups after blinatumomab treatment. We detected a decrease in the amount of *TNFSF4* expression in RS4;11. One explanation is that some RS4;11 cells already entered an apoptotic stage and had an aberrant transcriptome. At the same time, the SUP-B15 cells did not show significant changes because they are less sensitive to the blinatumomab treatment (Additional file 1, Fig. S10).

Furthermore, the distribution of *TNFSF4* expression in B-ALL patients was analyzed using a publicly available large-cohort data, which included expression profiles from BMMC samples from 576 B-ALL patients (Microarray Innovations in LEukemia study, GEO accession: GSE13204). The density curve of TNFSF4 expression was fitted to a Gaussian model and a Gaussian mixture model by an Expectation Maximization (EM) method, respectively. The plot showed that the Gaussian model may not fit the density curve well and that the null hypothesis of similarity between the two distributions was rejected (*p* < 0.05, Kolmogorov-Smirnov test, Additional file 1, Fig. S11A). The two-component Gaussian mixture model (component-1: μ = 0.436, σ2 = 0.019, mixing probability = 70.80%; component-2: μ = 0.650, σ2 = 0.004, mixing probability = 29.20%,) was a better fit for *TNFSF4* expression (*p* > 0.05, Kolmogorov-Smirnov test, Additional file 1, Fig. S11B). As it is shown in Fig. 5c, *TNFSF4* expression in patients with B-ALL had a bimodal distribution with nearly 34% of B-ALL patients expressing relatively high levels of *TNFSF4*.

In order to determine the impact of upregulation of TNRFSF4/TNFSF4 signaling on the cytolytic activity of blinatumomab, we used a recombinant human TNFSF4 protein in the cytotoxicity model. At first, PBMCs were co-cultured with target cells for 16 or 48 h at a ratio of 10:1 in the presence of the recombinant human TNFSF4 protein. No significant additive effects were found in the sensitive cell line RS4;11 by adding TNFSF4, as its baseline of blinatumomab-directed lysis was already very high (data not shown). Increased blinatumomab-directed lysis of the less sensitive SUP-B15 cells was observed after 48 but not 16 h of co-culturing, suggesting that the effect of TNFSF4 to the cytolytic activity is better detected in the long-time co-culturing condition (Additional file 1, Fig. S12). With an optimized assay condition of 1:2 effector-to-target cell ratio and 5 days of co-culturing, the recombinant human TNFSF4 protein significantly increased blinatumomab-directed lysis of the less sensitive SUP-B15 cells in a dose-dependent manner (Fig. 5d). This demonstrates that TNFSF4 was able to enhance the cytolytic capability of blinatumomab-activated T cells.

## Discussion

In our study, a single-cell transcriptome analysis allowed for the characterization of different T cell subpopulations and blinatumomab-activated clusters, as well as for the delineation of transcriptional changes in blinatumomab-activated T cells, and the identification of co-stimulatory ligand expression in blinatumomab-treated B-ALL cells. The results of this study shed light into the mechanisms underlyingthe target cell-dependent T cell responses induced by blinatumomab, as well as the potential mechanisms underlying the responses or resistance of B-ALL patients to blinatumomab treatment. These findings indicate that TNFRSF4 may serve as a potential target for combination therapy with blinatumomab to treat patients with B-ALL.

The functions of different T cell populations in blinatumomab-mediated cytotoxicity are a topic of active discussion in the field. Previous studies have shown that T_EM_ cells, not naïve T cells, T_CM_ cells, or CD45RA+ T_EM_ cells, account for blinatumomab activity [14, 24, 30, 31]. However, other studies showed that blinatumomab-mediated cytotoxicity was accomplished by various T cell populations, including Tregs [33, 34]. Single cell transcriptomics allowed a comprehensive and precise analysis of the impact of different T cell populations on blinatumomab-mediated cytotoxicity. We found that CD8+ T_EM_ cells, CD4+ T_CM_ cells and naïve T cells became activated and cytolytic after blinatumomab treatment. Blinatumomab-activated CD8+ T_EM_ cells were more cytolytic than other cells. In addition, blinatumomab-activated Tregs became strongly suppressive after activation.

Interestingly, it was previously reported that there is no correlation between the response to blinatumomab therapy and the absolute numbers of total T cells, CD4+ T cells, CD8+ T cells, naïve T cells, effector cells and proliferative T cells [33]. However, based on our single-cell transcriptome assay, we not only found a higher frequency of activated CD8+ T_EM_ cells, CD4+ T_CM_ cells and naïve T cells within the total T cell population, but also a higher frequency of activated Tregs among total Tregs, which were correlated with stronger blinatumomab-mediated cytotoxicity (Fig. 2d). These results suggest that the overall balance in cytolytic functions and immune suppressive functions, but not the frequency of certain cell population, influences the outcome of blinatumomab treatment. Therefore, the ratio of cytolytic and Tregs cells in patients may be correlated with the response to blinatumomab therapy. This result is partially consistent with a previous finding that the frequency of Tregs (CD4 + CD25^high^FOXP3+) among total T cells determines the outcome of blinatumomab therapy in patients with B-ALL [33].

Despite the clinical benefit of blinatumomab treatment, certain patients still fail to respond to blinatumomab therapy. During blinatumomab-mediated cytotoxicity, T cells and tumor cells form immune synapses via blinatumomab to initiate T cell activation. The ligands in the tumor cells bind to the co-signaling receptors on T cells and modulate T cell responses after activation. Previous studies have shown the importance of the co-inhibitory receptors PD-1 and CTLA4 for the cytolytic activity of blinatumomab [21, 26, 27]. Currently, a phase I trial combining blinatumomab with inhibitors of the co-inhibitory receptors PD-1 and CTLA4 is ongoing. However, not all patients benefit from this combination therapy [29], and multiple mechanisms may account for the resistance to blinatumomab therapy. A recent study in 44 adult B-ALL patients suggested that the intrinsic characteristics of tumors, like CD19 loss, may be responsible for blinatumomab resistance [61]. However, in our study, as well as on the CCLE database, no significant differences were found between the expression of *CD19* in RS4;11 and SUP-B15 (Additional file 1, Fig. S13A-B). Similarly, the expression of *CD19* did not significantly differ between bone marrow samples from those two patients (Additional file 1, Fig. S14). From the whole-transcriptome perspective, the differential gene expression profiles of RS4;11 and SUP-B15 were highly comparable (R^2^ = 0.9) before and after blinatumomab treatment (Additional file 1, Fig. S15). We analyzed the enriched pathways of differentially expressed genes between RS4;11 and SUP-B15, and found that the MHC class I antigen processing and presentation pathway was enriched in SUP-B15 cells (data not shown). However, T cell activation induced by blinatumomab is MHC-independent. We also compared the gene expression changes in RS4;11 and SUP-B15 cells after blinatumomab treatment and found that interferon-induced genes were up-regulated in RS4;11 cells, but not in SUP-B15 cells. This is an indication of stronger T cell responses to RS4;11 cells (data not shown). In this study, the limited PBMCs or BMMCs samples from only one healthy donor and two patients were used in single-cell transcriptome analysis, which may not be fully representative of the patient population. Thus, a larger cohort may need to be used in the future in order to generalize the findings.

It is believed that failure in some patients to respond to immunotherapy is the result of inadequate T cell activation that requires co-stimulatory signaling after T cell receptor signaling [62]. In agreement with these observations, few T cells were fully activated in the SUP-B15 group, which resulted in poor blinatumomab-directed lysis of SUP-B15 cells (Fig. 2d). Moreover, the ligands of the co-stimulatory signaling pathways are rarely present in tumor cells [62]. In this study, we found that a higher expression of *TNFSF4*, the ligand of the co-stimulatory receptor TNFRSF4, was present among the sensitive target cells RS4;11. Additionally, leukemia blast cells of patients with B-ALL showed differentiated *TNFSF4* expression, with only 34% of patients highly expressing *TNFSF4*. This suggests that the lack of TNFRSF4/TNFSF4-mediated co-stimulatory signaling may play a role in resistance to blinatumomab therapy.

TNFRSF4 belongs to the next generation of immune therapeutic targets in the field of oncology. Stimulation with TNFSF4 enhances the proliferation, survival and expression of effector factors in different T cell populations. By contrast, TNFRSF4/TNFSF4 signaling impairs the immune suppressive ability of Tregs [63–66]. Moreover, co-stimulatory signaling transduction by TNFRSF4 has been designed in the third-generation CARs. Accordingly, CARs containing CD28 and TNFRSF4 co-stimulation induced superior CCR7(−) T cell survival and lower IL-10 secretion than the CD28-only second-generation CARs [67–69]. In this study, we found that *TNFRSF4* was highly expressed on blinatumomab-activated cells, raising the possibility to preferentially target *TNFRSF4*. Indeed, the recombinant human TNFSF4 protein increased the cytolytic activity of blinatumomab ex vivo. Therefore, TNFRSF4 might be a potential target for the combination therapy to improve blinatumomab-activated T cell functions and increase clinical benefits to B-ALL patients.

## Conclusions

The analysis of single cell transcriptomes reveals responses of different T-cell populations and the mechanism behind target cell-dependent T-cell activation in response to blinatumomab treatment. The co-stimulatory receptor TNFRSF4 is upregulated in blinatumomab-activated T cells. B-ALL cell expression of TNFSF4 is positively correlated with blinatumomab sensitivity and rhTNFSF4 stimulates activity of blinatumomab. Further studies are needed in the future to validate the role of TNFSF4 in blinatumomab responsiveness.

## Methods

### Blinatumomab-mediated cytotoxicity assay

The blinatumomab-mediated cytotoxicity assay was developed upon, and modified from, previous methods [23, 33, 34]. Briefly, we used frozen healthy Peripheral blood mononuclear cells (PBMCs, HemaCare, #PB009C-2) and the B-ALL cell lines RS4;11 (ATCC, CRL-1873) and SUP-B15 (ATCC, CRL-1929) as effector and target cells, respectively. The frozen PBMCs were commercial products and collected by vendor with a limited cell number from each donor. Thus, the PBMCs that were used in the different assays came from different donors. The target to effector cell ratio and blinatumomab concentration were titrated for different experiments. For the cell line cytotoxicity assay, the target cells were stained using the CellTrace™ Far Red Cell Proliferation Kit (Invitrogen, # C34564) and then co-cultured with PBMCs for 16 or 48 h at an effector-to-target cell ratio of 10:1 and in the presence of blinatumomab. For the TNFSF4 assay, titration started from an E:T ratio of 10:1 for 16 and 48 h. In order to observe a significant dose-response effect of TNFSF4, the PBMCs were co-cultured with target cells for 5 days at a ratio of 1:2 in the presence of recombinant human TNFSF4 protein (R&D system, #1054-OX-010). The cell mixture was then stained using a L/D staining solution (Zombie Aqua Fixable viability kit, Biolegend, #423101) for 30 min, resuspended in a flow cytometry staining buffer (eBioscience, #00422226), and then 10000 target cells were analyzed on a BD LSR Fortessa™ X-20. Specific lysis was calculated according to the following equation: % specific lysis = (proportion of live target cells in sample - proportion of live target cells in control sample) / (1 - proportion of live target cells in control sample) × 100.

### Single-cell isolation and RNA sequencing

PBMCs were co-cultured with target cells for 16 or 48 h at a ratio of 10:1 in the presence of either 0 or 0.1 ng/mL blinatumomab. B-ALL patient PBMCs and bone marrow mononuclear cells (BMMCs) (*N* = 3, Proteogenex, #ALL205BM, #ALL207BM, #ALL207L) were cultured for 16 h in the presence of either 0 or 10 ng/mL blinatumomab. The cells were harvested and resuspended to 1 × 10^6^ cells/mL with DPBS containing 1% FBS after removing cell debris and large clumps using a 40-μm cell strainer (StemCell, #352235). Subsequently, single cells were isolated on a 10X Genomics Chromium Controller using the Chromium Single Cell 3′ Reagent Kit v2 for the cell line model, or Chromium Single Cell V(D)J Reagent Kits for the patient derived model. After the isolation of single cells, the cDNA was synthesized, and the sequencing library was constructed. High-throughput scRNA-seq data was generated using an Illumina HiSeq PE150. All samples were purchased from HemaCare and Proteogenex. We obtained informed consent in accordance with the relevant institutional ethical review boards.

### Preprocessing of the single-cell RNA-Seq data

The raw sequencing data (FASTQ file) of each sample was first quality-controlled using *FastQC* and fed into the 10X’s *cellranger count* pipeline [70] for alignment, filtering, barcode counting and unique molecular identifiers (UMI) counting. Barcodes with a total UMI count between 100 and 1000 were classified as cell-containing at a false discovery rate of 0.1% using the Monte Carlo permutation test from the *DropletUtils* package [71, 72]. Barcodes with an UMI count of more than 1000 were retained as cell-containing. The two groups of cell-containing barcodes (*N* = 76,809) were then pooled together for subsequent analysis. Next, we removed low-quality cells, such as damaged or dying cells, by assessing several quality control metrics (specifically, UMI count, gene count and mitochondrial proportion) as provided by the *scater* R package [73]. Low-quality cells (*N* = 3970) were filtered using a cutoff of more than 3 median absolute deviations (MADs). Cells (*N* = 64,613) predicted to be in the G1 phase using the classifier provided in the *scran* package [74] were kept for downstream analysis. The deconvolution method from the *scran* package [74] was used for data normalization. Batch effects were corrected by a mutual nearest neighbor-based method available in the *scater* package [75].

### Unsupervised classification of cell type

Data dimensionality was reduced using principal component analysis. Based on the Euclidean distance between cells in the first 20 principal components, a *k*-nearest neighbor graph was built and the Louvain algorithm was used to calculate modularity, as available in the *Seurat* R package [76]. Here, the resolution parameter, which sets the granularity of the subsequent cluster extraction, was chosen to optimally segregate all cells and T cells in the two datasets in our analysis, respectively. For visualization purposes, we embedded these 20 principal components in a two-dimensional space using the t-SNE approach. Differential gene expression and marker detection was performed using the two-part generalized linear model implemented in *MAST* [77]. The parameters for marker detection were set as, only.pos = TRUE, min.pct = 0.25 and logfc.threshold = 0.25.

### Temporal trajectory analysis

To unveil the state transition graph of blinatumomab treatment-induced T-cell subtypes, the reversed graph embedding algorithm was used to reconstruct single-cell trajectories in a fully unsupervised manner. The cells were aligned to a latent temporal axis and assigned pseudo-time units using the *Monocle2* R package (version 2.8.0) on high variable genes. The genes that significantly changed along the pseudo-temporal trajectory were identified using the test statistics implemented in *Monocle2* with a *q*-value cutoff of < 0.01 [78]. Gene expression dynamics were visualized as a function of pseudo-time with the *CellTrails* R package [79].

### Quantitative real-time PCR

Total RNA from cells belonging to the RS4;11 and SUP-B15 groups was extracted using anRNeasy Plus Mini Kit (Qiagen). The cDNA was synthesized (SuperScript IV FirstStrand Synthesis System, Thermofisher) and quantitative real-time PCR (qPCR) was performed using the Power SYBR™ Green PCR Master Mix (Invitrogen) on a StepOnePlus Real-Time PCR system (Applied Biosystems). The following primers were used for qPCR: *TNFSF4*, forward 5′-CCTACATCTGCCTGCACTTCTC-3′, reverse 5′-TGATGACTGAGTTGTTCTGCAC C-3′; *GAPDH*, forward 5′-GTCTCCTCTGACTTCAACAGCG-3′, reverse 5′-ACCACC CTGTTGCTGTAGCCAA-3′.

### Curve fitting for *TNFSF4* expression in B-ALL patients

The relative expression data of *TNFSF4* (probe set id: 207426_s_at) in the bone marrow samples of 576 patients with acute B-lymphoblastic leukemia were retrieved from Microarray Innovations in the LEukemia (MILE) study (GEO accession: GSE13204). In order to better illustrate the distribution of *TNFSF4* expression in patients, a Gaussian mixture model was used to fit its density curve with the Mclust package. The parameters for each model were estimated by an Expectation Maximization method. The best model was selected from a combination of parameter pairs between mixing components number (G, from 1 to 9) and variance equality (E: equal variance, V: different variance) according to the Bayesian information criterion (BIC) value. The model is a classical Gaussian distribution (also known as normal distribution), when G equals to 1. To evaluate the fitness, the distribution of each model was compared with the density curve of *TNFSF4* expression by the Kolmogorov-Smirnov test with the null hypothesis that the two distributions are similar. If the *p*-value was less than 0.05, the null hypothesis was rejected.

### Statistics and reproducibility

For Figs. 1b and 5b&d, the experiments were performed a minimum of three times to ensure independent experimental replication. Statistical analyses (*n* = 3 or more) were performed using the GraphPad Prism 7 and a two-sided paired Student’s t-test. Data are shown as the mean ± SEM. Calculated *P*-values are indicated as non-significant (ns), *P < 0.05, **P < 0.01 and ***P < 0.001.

## Supplementary Information

**Additional file 1: Figure S1.** Establishment and clustering of cell line model for blinatumomab-induced cytotoxicity. (a-b) Specific lysis curves of target cells after treatment with serial concentrations of blinatumomab for (a) 16 and (b) 48 h. The green and blue lines represent RS4;11 and SUP-B15 cells as the target cells, respectively. Data are mean ± SD of three biological replicates. *P < 0.05; **P < 0.01; ***P < 0.001; Two-way ANOVA analysis. (c) A t-SNE projection of all the single cells from cell line model. Different samples are shown in different colors. (d) Violin plots showing the expression of well-known marker genes to define the cell type in 5 main clusters. **Figure S2**. Removal of the cluster composed of doublets. (a) A t-SNE projection of all T cells from the cell line model dataset. Different clusters are shown in different colors. (b) Violin plot showing the numbers of genes detected in single cells from the 18 T cell clusters. (c) Violin plot showing the numbers of unique molecular identifiers (UMI) detected in single cells from the 18 T cell clusters. **Figure S3.** Identification of CD8+ and CD4+ T cell subtypes. (a) Expression levels of *CD4* and *CD8* in all single T cells as illustrated in t-SNE plots in red and green, respectively. (b) A t-SNE projection of all T cells from cell line model. Clusters are colored based on CD4/*CD8* expression ratio, where all cells in a given cluster are assigned the same average value. **Figure S4.** Identification and analysis of activated Tregs. (a) Violin plots showing the expression of blinatumomab activation marker genes in 17 T cell clusters. (b) A t-SNE projection of all Tregs identified in Fig. 2a, which contains 3 subclusters in different colors. The identity of each cluster was determined based on the signature genes in each cluster. (c) Heatmap showing the expression of the top 20 signature genes in each Treg subcluster of single cells. (d) The proportion of each cluster in the untreated (RU-16 h, RU-48 h, SU-16 h, and SU-48 h) and blinatumomab-treated (RT-16 h, RT-48 h, ST-16 h, and ST-48 h) groups. **Figure S5.** Enrichment of blinatumomab-responsive clusters. The percentages of TC6-CD4+ Naïve T-STAT1 and TC8-CD4 + TCM-IFIT3 cells among total T cells in each sample and the percentage of IFN-Tregs among total Tregs in each sample. RS4;11-16 h represents RU-16 h and RT-16 h. RS4;11-48 h represents RU-48 h and RT-48 h. SUP-B15-16 h represents SU-16 h and ST-16 h. SUP-B15-48 h represents SU-48 h and ST-48 h. **Figure S6.** The proportion of T cells for each T cluster in separate view. **Figure S7.** T-cell trajectories of CD8+ and CD4+ T cells. (a-b) The trajectory of (a) all CD8+ T-cell clusters, except MAIT cells, and (b) CD4+ T cell clusters, except TC6, TC8, and TC12, in a 2D state space defined by Monocle 2. Each point represents a single cell and each color represents a cluster. (c-d) Expression of genes associated with activation in select (c) CD8+ and (d) CD4+ T cells ordered based on pseudotime. Each point represents a single cell and each color represents a cluster. The same colors were used here as in (c) Fig. S6a and (d) Fig. S6b. **Figure S8.** Analysis of cell types in patient derived model. (a) A t-SNE projection of all cells in patient derived model with the 5 main clusters in different colors. The identity of each cluster was determined based on the signature genes of each cluster. Cluster PC0: tumor cells; Cluster PC1: tumor cells; Cluster PC2: T cells and NK cells; Cluster PC3: B cells; Cluster PC4: red cells. (b) A t-SNE projection of all cells from patient derived model. Cells were colored based on expression level of *CD3E*. (c) A t-SNE projection of all T cells from patient derived model. Cells were colored based on expression level of select genes. (d) Matrix showing the number of the top 20 signature genes shared by each cluster in cell line model samples and each cluster in patient derived model. (e) Bar plot of the expression of *TNFRSF4*, *TNFRSF18* and *LAG3* in clusters PTC0-CD8+ T_EM_ and PTC2-CD8+ Activated T. The y-axis showed the log2 value of the expression value. Δ represented the log2 value of the fold change. **Figure S9.** Enrichment of blinatumomab-activated clusters in patient samples. The percentages of PTC2 (a) and PTC6(b) cells among total T cells in 205BM and 207BM samples. **Figure S10.** Expression levels of TNFSF4 in the RS4;11 and SUP-B15 before and after Blinatumomab treatment for 48 h. T test was used to calculate the statistical significance for each comparison. **Figure S11.** The fitting curve of TNFSF4 expression in B-ALL patient. (a) Quantile-quantile (Q-Q) plot for 1-component Gaussian distribution. The *P* value is for the Kolmogorov-Smirnov test. If P value is less than 0.05, the null hypothesis that the two distribution are similar was rejected. A model is better if the points are closer to the diagonal line. (b) Q-Q plot for 2-component Gaussian mixture model. **Figure S12.** Specific lysis of SUP-B15 cells after treatment with 0.1 ng/mL blinatumomab and recombinant human TNFSF4 protein for 16 h or 48 h. The experiment was conducted in three independent replicates. *P < 0.05; **P < 0.01; ***P < 0.001; Student’s t-test. **Figure S13.** The CD19 expression in RS4;11 and SUP-B15. (a) The CD19 expression profile in RS4;11 and SUP-B15 in CCLE database. Grey points represent other cell lines in CCLE (b) Violin plots showing the expression of CD19 in RS4;11 and SUP-B15 cells from the sc-RNAseq dataset. **Figure S14.** Violin plot of relative CD19 expression in tumor cells from samples 205BM and 207BM based on scRNA-Seq data. **Figure S15**. The whole-genome gene expression comparison between RS4;11 and SUP-B15 cell line before and after Blinatumomab. **Table S1.** Sample information.

**Additional file 2: Table S2.** Signature genes in each T cell subcluster in cell line model.

**Additional file 3.** Detailed description of cell clustering and definition in the two models.

**Additional file 4: Table S3.** Differentially expressed genes induced by blinatumomab in cell line model.

**Additional file 5: Table S4.** Signature genes in each T cell subcluster in patient derived model.

**Additional file 6: Table S5.** Differentially expressed genes induced by blinatumomab in patient derived model.

## Data Availability

The single cell RNA-seq datasets generated during the current study are available in the Sequence Read Archive (SRA) repository (Accession Number: SRR13518689, SRR13518690, SRR13518691, SRR13518692, SRR13518693, SRR13518694, SRR13518695, SRR13518696, SRR13518697, SRR13518698, SRR13518699, SRR13518700, SRR13518701 and SRR13518702 in BioProject PRJNA694543). The expression profiles of BMMC samples from 576 B-ALL patients in Microarray Innovations in LEukemia study are available in the Gene Expression Omnibus (GEO) repository (Accession Number: GSE13204). The CD19 expression data of RS4;11 and SUP-B15 cell line are from the Broad Institute Cancer Cell Line Encyclopedia (CCLE) website (https://portals.broadinstitute.org/ccle/). Other relevant data are presented in supplement of this article.

## Abbreviations

B-ALL: Acute B cell lymphoblastic leukemia
FDA: Food and Drug Administration
ALL: Acute lymphoblastic leukemia
CAR: Chimeric antigen receptor
BiTE: Bispecific T cell engager
scRNA-seq: Single-cell RNA-Sequencing
T_EM_: Effector memory T cell
T_CM_: Central memory T cell
Treg: Regulatory T cell
PBMC: Peripheral blood mononuclear cells
BMMC: Bone marrow mononuclear cells
UMI: Unique molecular identifiers
qPCR: Quantitative real-time PCR
IFN: Interferon
DEG: Differentially expressed gene
t-SNE: T-distributed stochastic neighbor embedding
DNT: Double negative T cell
NKT: Natural killer T cell
RU-16 h: Untreated RS4;11 cells at 16 h
RU-48 h: Untreated RS4;11 cells at 48 h
SU-16 h: Untreated SUP-B15 cells at 16 h
SU-48 h: Untreated SUP-B15 cells at 48 h
RT-16 h: Blinatumomab-treated RS4:11 cells at 16 h
RT-48 h: Blinatumomab-treated RS4;11 cells at 48 h
ST-16 h: Blinatumomab-treated SUP-B15 cells at 16 h
ST-48 h: Blinatumomab-treated SUP-B15 cells at 48 h
207 L-B0: PBMCs from patient #207 cultured without blinatumomab for 16 h
207 L-B50: PBMCs from patient #207 cultured with blinatumomab for 16 h
205BM-B0: BMMCs from patient #205 cultured without blinatumomab for 16 h
205BM-B50: BMMCs from patient #205 cultured with blinatumomab for 16 h
207BM-B0: BMMCs from patient #207 cultured without blinatumomab for 16 h
207BM-B50: BMMCs from patient #207 cultured with blinatumomab for 16 h
EM: Expectation Maximization
GEO: Gene Expression Omnibus
MAD: Median absolute deviation
