# Supplementary material for: Blinatumomab-induced T cell activation at single cell transcriptome resolution
Source: BMC Genomics. 2021 Mar 1;22:145. doi: 10.1186/s12864-021-07435-2 (PMC7923532; doi:10.1186/s12864-021-07435-2)
Supplement: Supplementary file 1 — Additional file 1: Figure S1. Establishment and clustering of cell line model for blinatumomab-induced cytotoxicity. (a-b) Specific lysis curves of target cells after treatment with serial concentrations of blinatumomab for (a) 16 and (b) 48 h. The green and blue lines represent RS4;11 and SUP-B15 cells as the target cells, respectively. Data are mean ± SD of three biological replicates. *P < 0.05; **P < 0.01; ***P < 0.001; Two-way ANOVA analysis. (c) A t-SNE projection of all the single cells from cell line model. Different samples are shown in different colors. (d) Violin plots showing the expression of well-known marker genes to define the cell type in 5 main clusters. Figure S2. Removal of the cluster composed of doublets. (a) A t-SNE projection of all T cells from the cell line model dataset. Different clusters are shown in different colors. (b) Violin plot showing the numbers of genes detected in single cells from the 18 T cell clusters. (c) Violin plot showing the numbers of unique molecular identifiers (UMI) detected in single cells from the 18 T cell clusters. Figure S3. Identification of CD8+ and CD4+ T cell subtypes. (a) Expression levels of CD4 and CD8 in all single T cells as illustrated in t-SNE plots in red and green, respectively. (b) A t-SNE projection of all T cells from cell line model. Clusters are colored based on CD4/CD8 expression ratio, where all cells in a given cluster are assigned the same average value. Figure S4. Identification and analysis of activated Tregs. (a) Violin plots showing the expression of blinatumomab activation marker genes in 17 T cell clusters. (b) A t-SNE projection of all Tregs identified in Fig. 2a, which contains 3 subclusters in different colors. The identity of each cluster was determined based on the signature genes in each cluster. (c) Heatmap showing the expression of the top 20 signature genes in each Treg subcluster of single cells. (d) The proportion of each cluster in the untreated (RU-16 h, RU-48 h, SU-16 h [file 12864_2021_7435_MOESM1_ESM.docx]

**Supplementary Materials**

**Blinatumomab-induced T Cell Activation at Single Cell Transcriptome Resolution**

Yi Huo^1,2#^, Zhen Sheng^1,2#^, Daniel Lu^3^, Daniel C. Ellwanger^3^, Chi-Ming Li^3^, Oliver Homann^3^, Songli Wang^3^, Hong Yin^2*^, and Ruibao Ren^1*^

^1^Shanghai Institute of Hematology, State Key Laboratory for Medical Genomics, National Research Center for Translational Medicine at Shanghai, Collaborative Innovation Center of Hematology, RuiJin Hospital affiliated to Shanghai Jiao Tong University School of Medicine, Shanghai, China;

^2^Amgen Asia R&D Center, Amgen Biopharmaceutical R&D (Shanghai) Co., Ltd., Shanghai, China;

^3^Genome Analysis Unit, Amgen Research, South San Francisco, Amgen Inc., California, United States

# Authors contributed equally

* Correspondence to: Ruibao Ren, [rbren@sjtu.edu.cn](mailto:rbren@sjtu.edu.cn); Hong Yin, [yinh@amgen.com](mailto:yinh@amgen.com).

**Supplemental Figures**


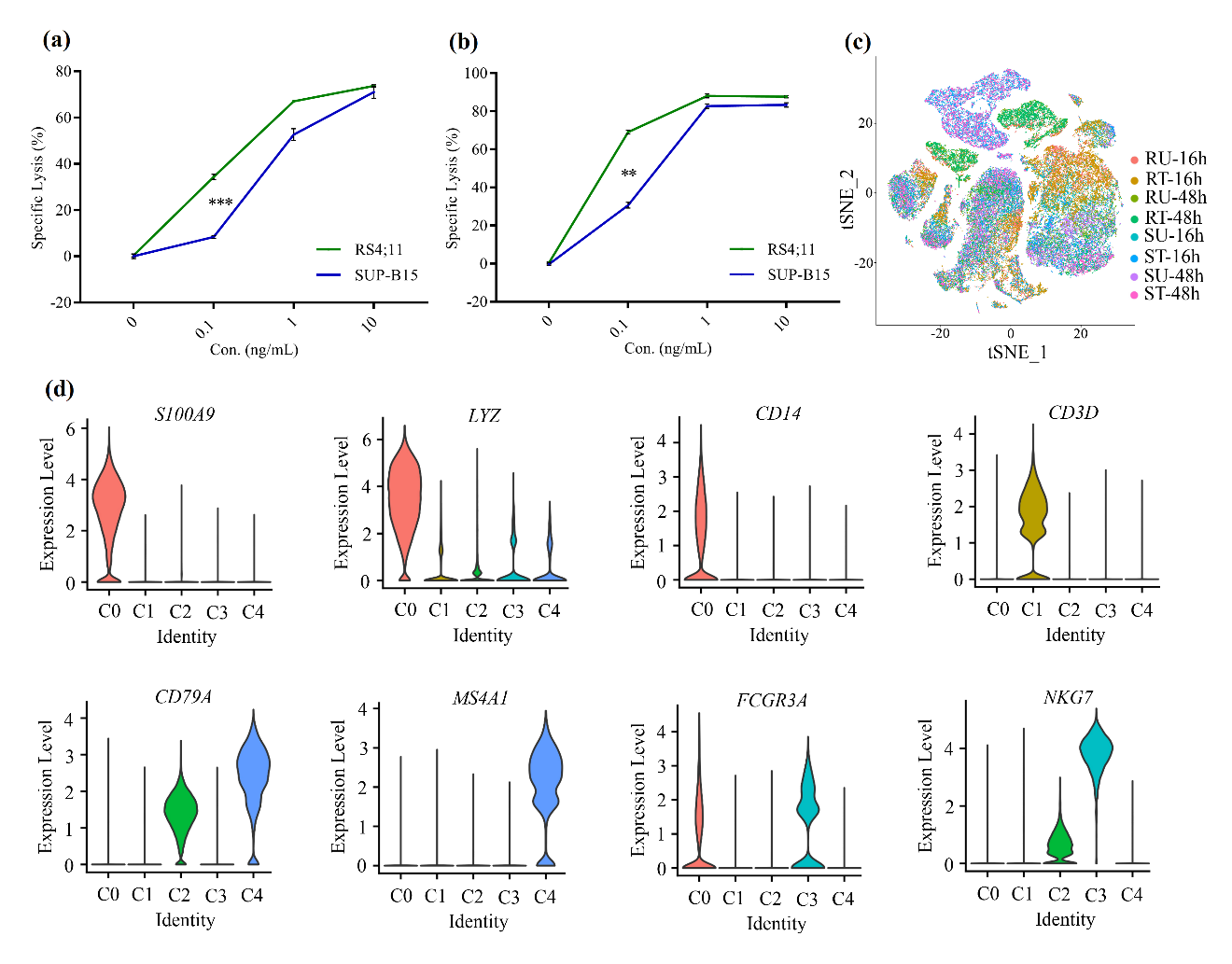


**Fig. S1.** Establishment and clustering of cell line model for blinatumomab-induced cytotoxicity. (a-b) Specific lysis curves of target cells after treatment with serial concentrations of blinatumomab for (a) 16 and (b) 48 h. The green and blue lines represent RS4;11 and SUP-B15 cells as the target cells, respectively. Data are mean ± SD of three biological replicates. *P < 0.05; **P < 0.01; ***P < 0.001; Two-way ANOVA analysis. (c) A t-SNE projection of all the single cells from cell line model. Different samples are shown in different colors. (d) Violin plots showing the expression of well-known marker genes to define the cell type in 5 main clusters.


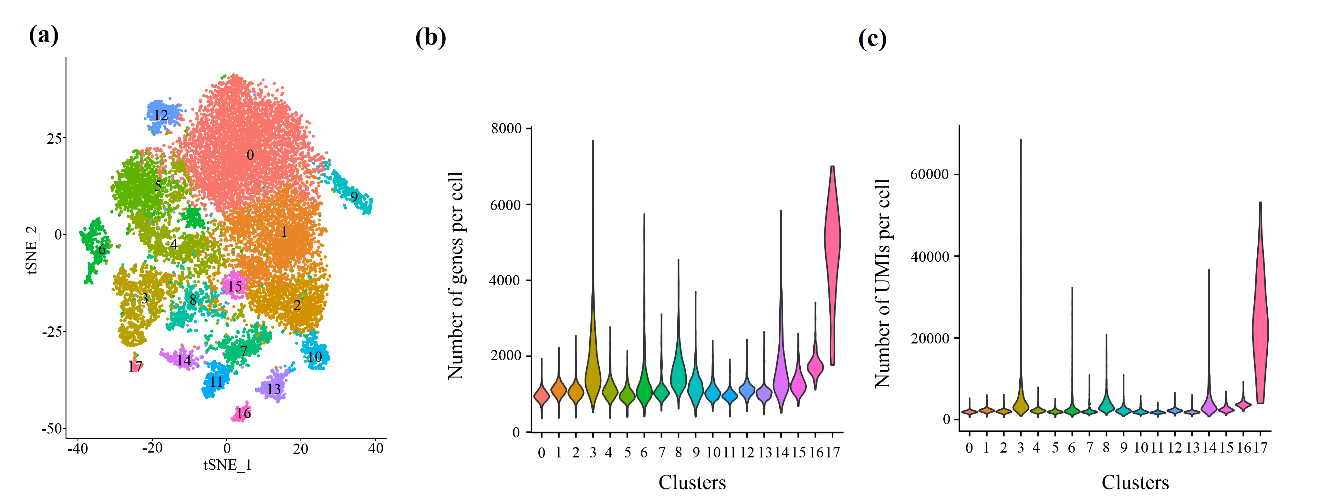


**Fig. S2.** Removal of the cluster composed of doublets. (a) A t-SNE projection of all T cells from the cell line model dataset. Different clusters are shown in different colors. (b) Violin plot showing the numbers of genes detected in single cells from the 18 T cell clusters. (c) Violin plot showing the numbers of unique molecular identifiers (UMI) detected in single cells from the 18 T cell clusters.


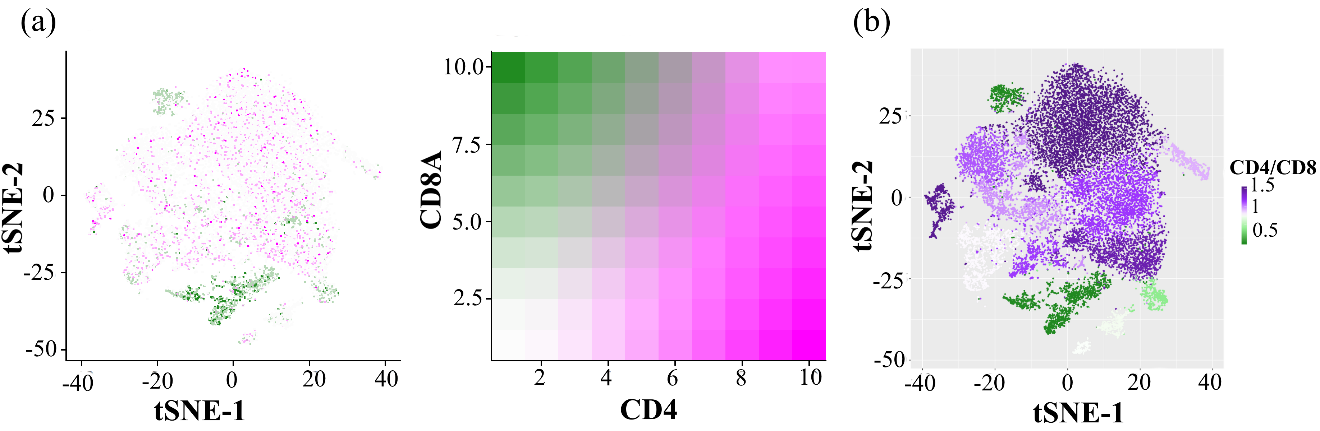


**Fig. S3.** Identification of CD8+ and CD4+ T cell subtypes. (a) Expression levels of *CD4* and *CD8* in all single T cells as illustrated in t-SNE plots in red and green, respectively. (b) A t-SNE projection of all T cells from cell line model. Clusters are colored based on CD4/*CD8* expression ratio, where all cells in a given cluster are assigned the same average value.


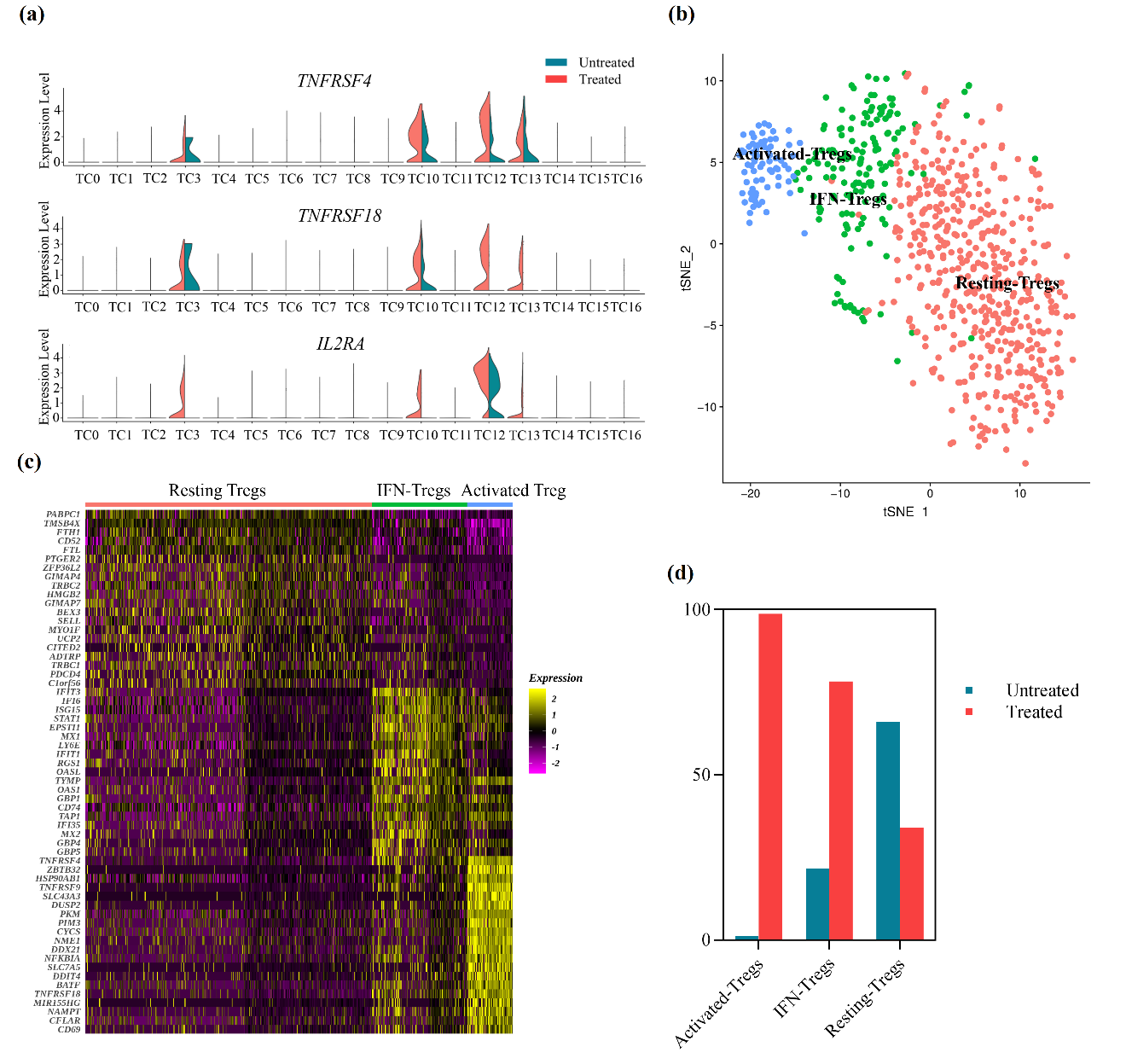


**Fig. S4.** Identification and analysis of activated Tregs. (a) Violin plots showing the expression of blinatumomab activation marker genes in 17 T cell clusters. (b) A t-SNE projection of all Tregs identified in Fig. 2A, which contains 3 subclusters in different colors. The identity of each cluster was determined based on the signature genes in each cluster. (c) Heatmap showing the expression of the top 20 signature genes in each Treg subcluster of single cells. (d) The proportion of each cluster in the untreated (RU-16h, RU-48h, SU-16h, and SU-48h) and blinatumomab-treated (RT-16h, RT-48h, ST-16h, and ST-48h) groups.


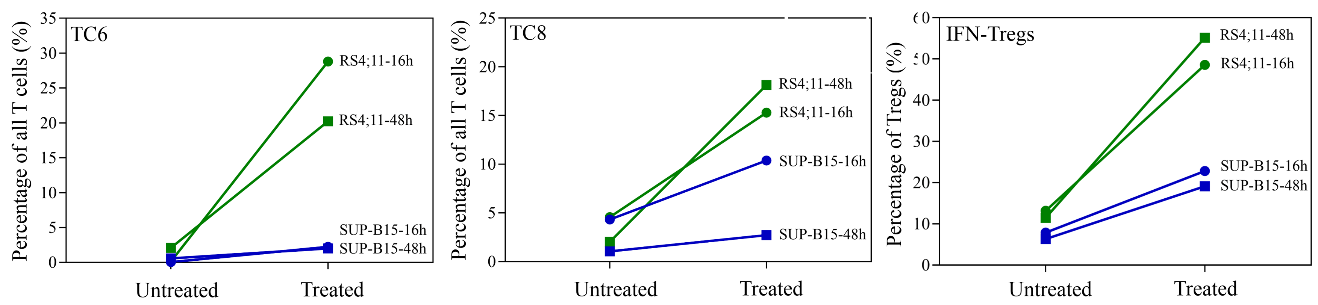


**Fig. S5.** Enrichment of blinatumomab-responsive clusters. The percentages of TC6-CD4+ Naïve T-STAT1 and TC8-CD4+TCM-IFIT3 cells among total T cells in each sample and the percentage of IFN-Tregs among total Tregs in each sample. RS4;11-16h represents RU-16h and RT-16h. RS4;11-48h represents RU-48h and RT-48h. SUP-B15-16h represents SU-16h and ST-16h. SUP-B15-48h represents SU-48h and ST-48h.


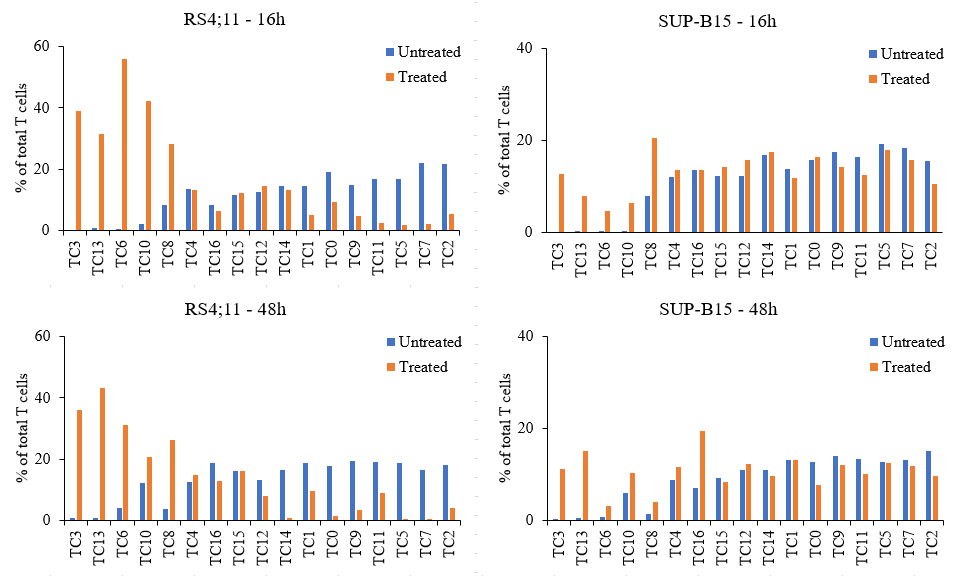


**Fig. S6. The proportion of T cells for each T cluster in separate view.**

**
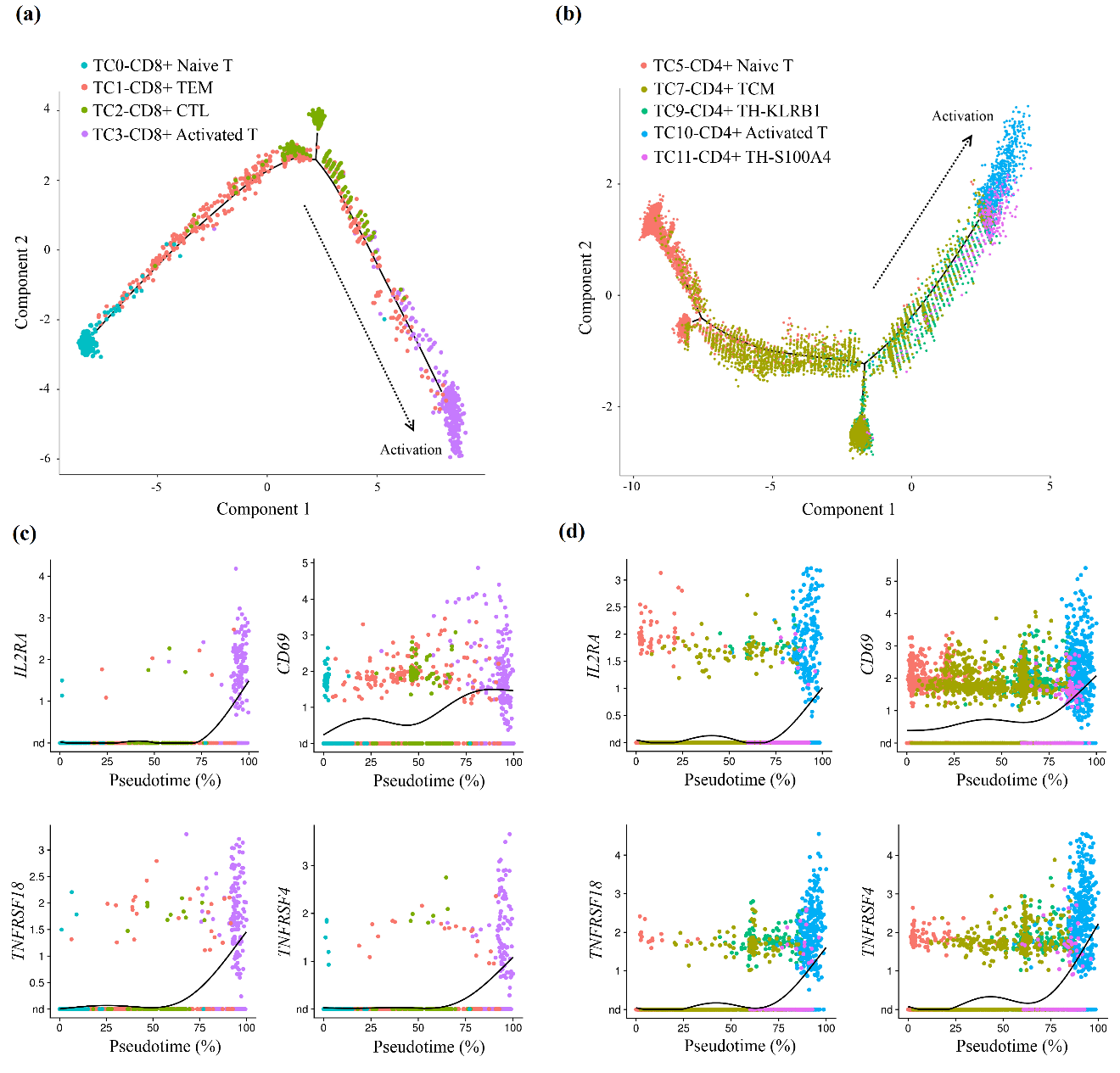
**

**Fig. S7.** T-cell trajectories of CD8+ and CD4+ T cells. (a-b) The trajectory of (a) all CD8+ T-cell clusters, except MAIT cells, and (b) CD4+ T cell clusters, except TC6, TC8, and TC12, in a 2D state space defined by Monocle 2. Each point represents a single cell and each color represents a cluster. (c-d) Expression of genes associated with activation in select (c) CD8+ and (d) CD4+ T cells ordered based on pseudotime. Each point represents a single cell and each color represents a cluster. The same colors were used here as in (c) Fig. S6a and (d) Fig. S6b.


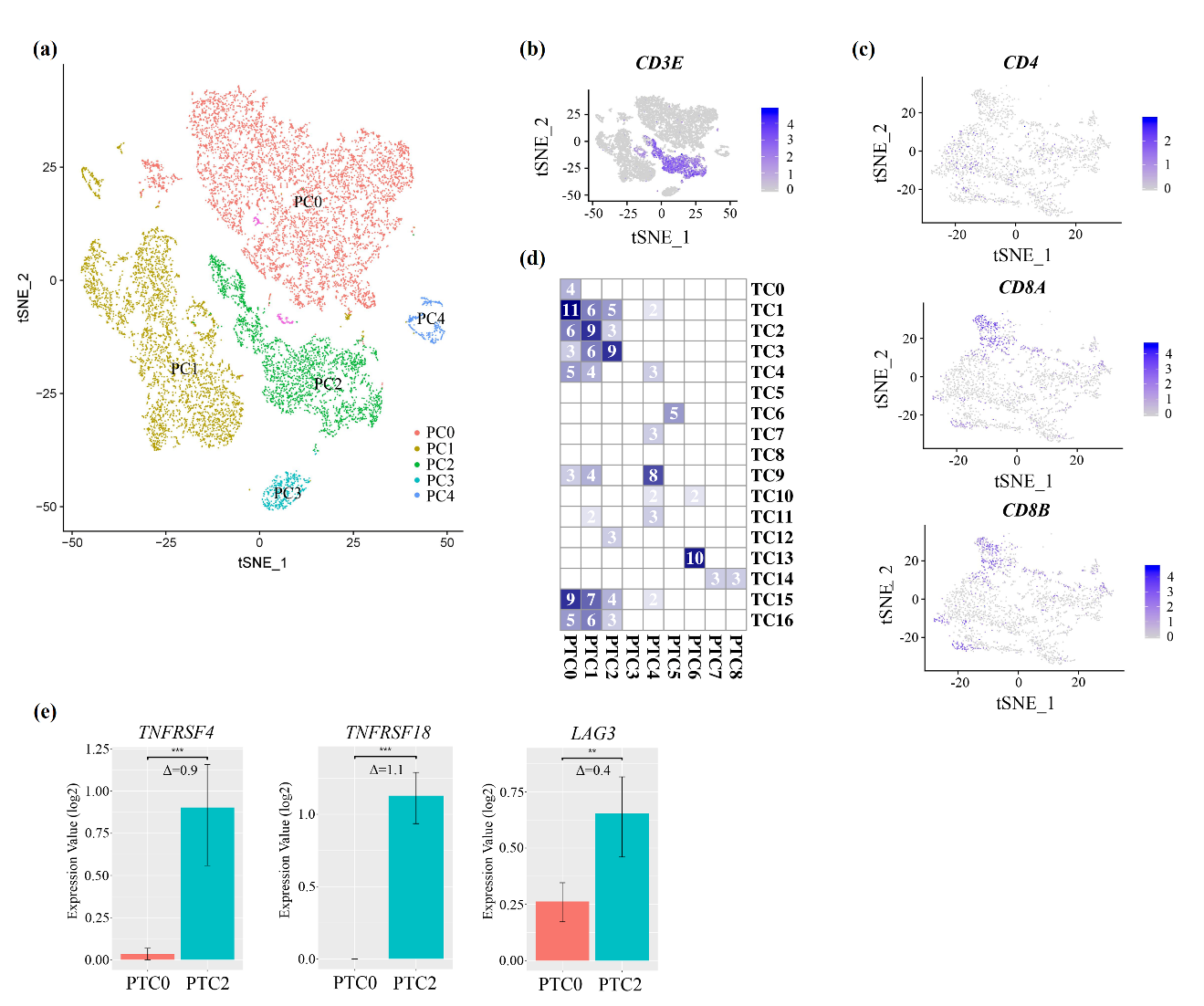


**Fig. S8.** Analysis of cell types in patient derived model. (a) A t-SNE projection of all cells in patient derived model with the 5 main clusters in different colors. The identity of each cluster was determined based on the signature genes of each cluster. Cluster PC0: tumor cells; Cluster PC1: tumor cells; Cluster PC2: T cells and NK cells; Cluster PC3: B cells; Cluster PC4: red cells. (b) A t-SNE projection of all cells from patient derived model. Cells were colored based on expression level of *CD3E*. (c) A t-SNE projection of all T cells from patient derived model. Cells were colored based on expression level of select genes. (d) Matrix showing the number of the top 20 signature genes shared by each cluster in cell line model samples and each cluster in patient derived model. (e) Bar plot of the expression of *TNFRSF4*, *TNFRSF18* and *LAG3* in clusters PTC0-CD8+ T_EM_ and PTC2-CD8+ Activated T. The y-axis showed the log2 value of the expression value. Δ represented the log2 value of the fold change.


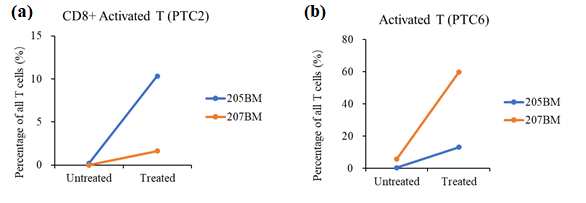


**Fig. S9.** Enrichment of blinatumomab-activated clusters in patient samples. The percentages of PTC2 (a) and PTC6(b) cells among total T cells in 205BM and 207BM samples.


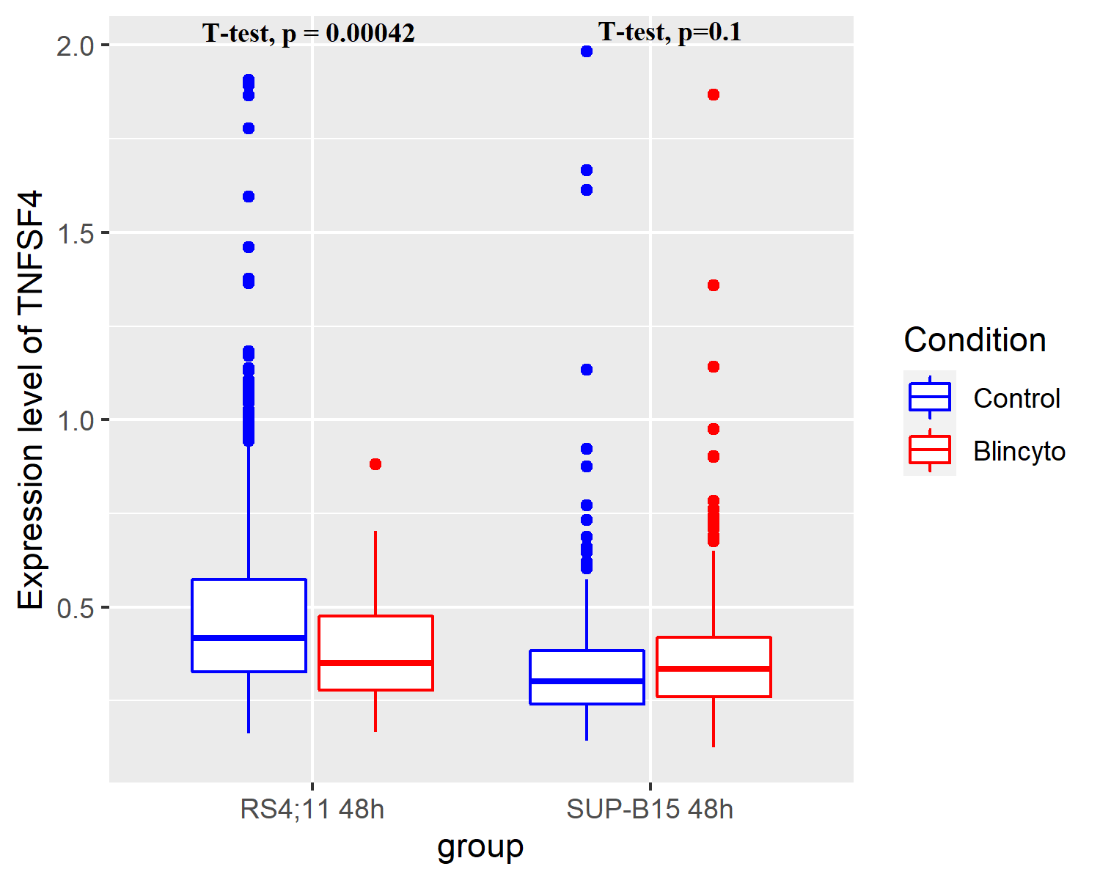


**Fig. S10.** Expression levels of TNFSF4 in the RS4;11 and SUP-B15 before and after Blinatumomab treatment for 48 h. T test was used to calculate the statistical significance for each comparison.


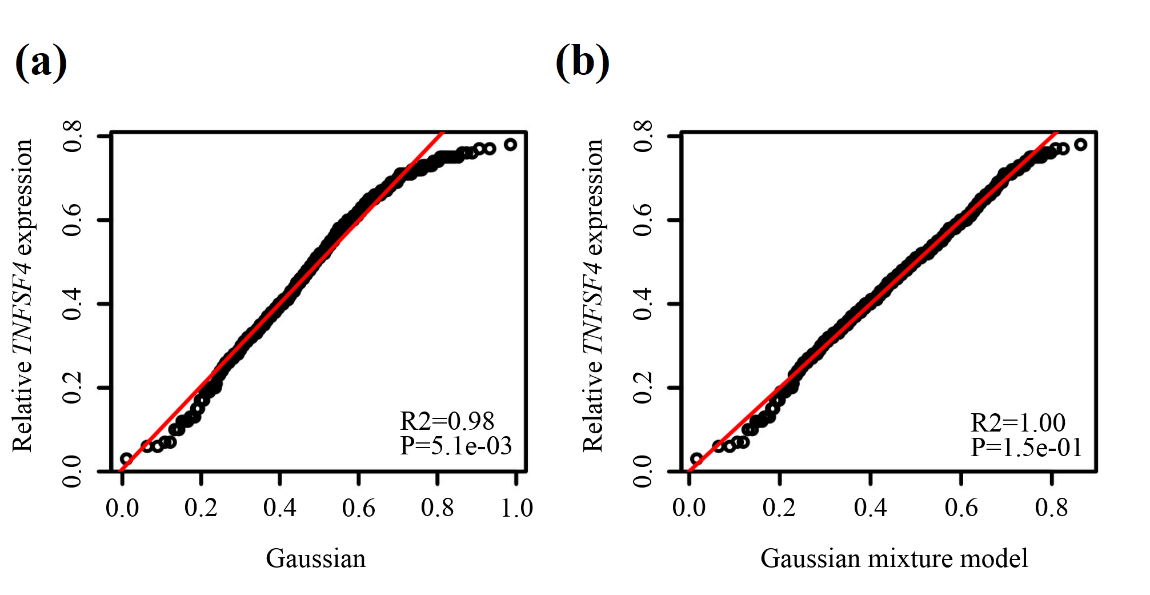


**Fig. S11.** The fitting curve of TNFSF4 expression in B-ALL patient. (a) Quantile-quantile (Q-Q) plot for 1-component Gaussian distribution. The P value is for the Kolmogorov-Smirnov test. If P value is less than 0.05, the null hypothesis that the two distribution are similar was rejected. A model is better if the points are closer to the diagonal line. (b) Q-Q plot for 2-component Gaussian mixture model.


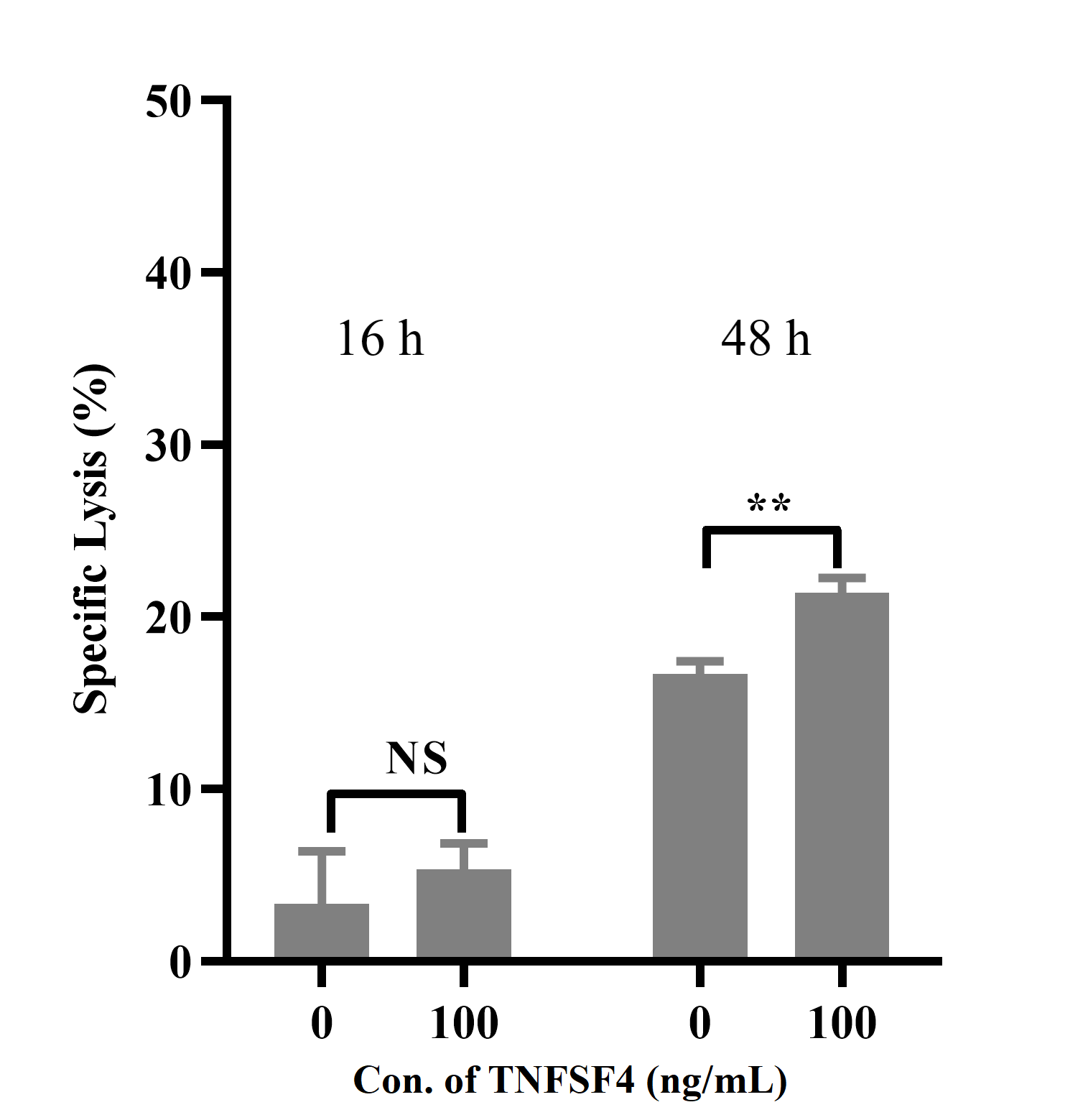


**Fig. S12.** Specific lysis of SUP-B15 cells after treatment with 0.1 ng/mL blinatumomab and recombinant human TNFSF4 protein for 16 h or 48 h. The experiment was conducted in three independent replicates. *P < 0.05; **P < 0.01; ***P < 0.001; Student's t-test.


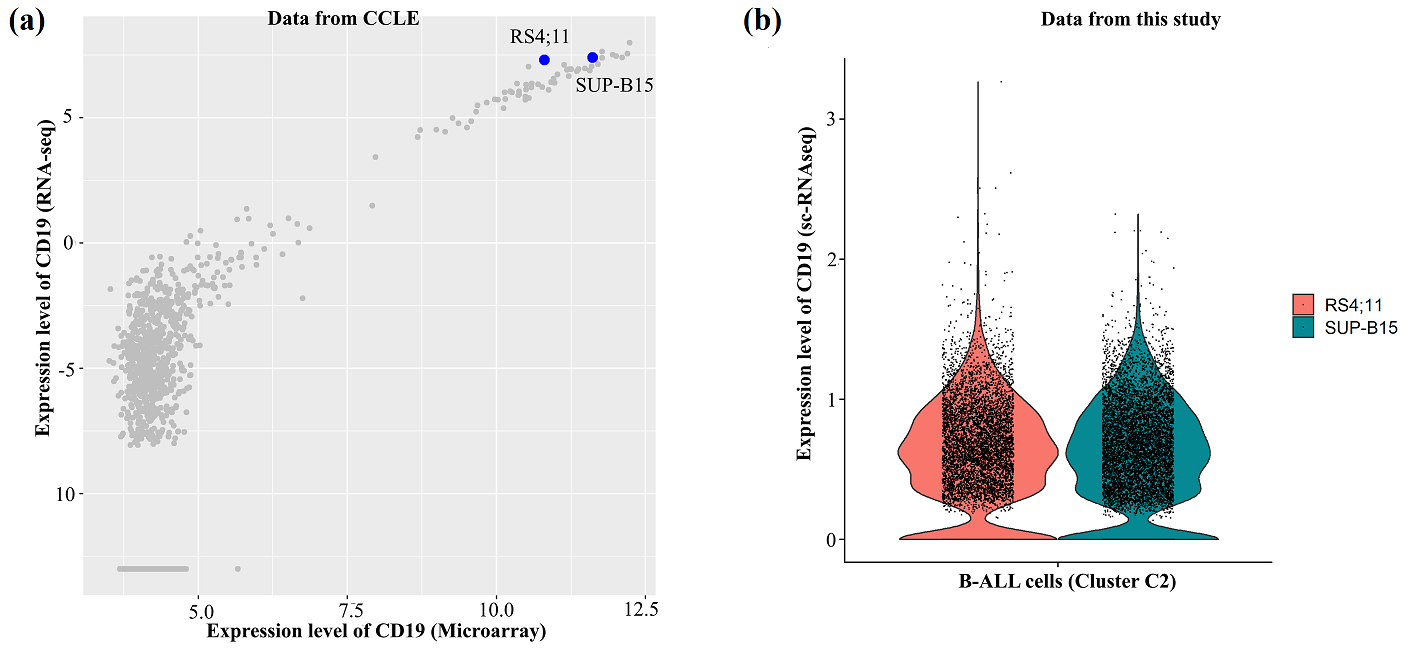


**Fig. S13.** The CD19 expression in RS4;11 and SUP-B15. (a) The CD19 expression profile in RS4;11 and SUP-B15 in CCLE database. Grey points represent other cell lines in CCLE (b) Violin plots showing the expression of CD19 in RS4;11 and SUP-B15 cells from the sc-RNAseq dataset.


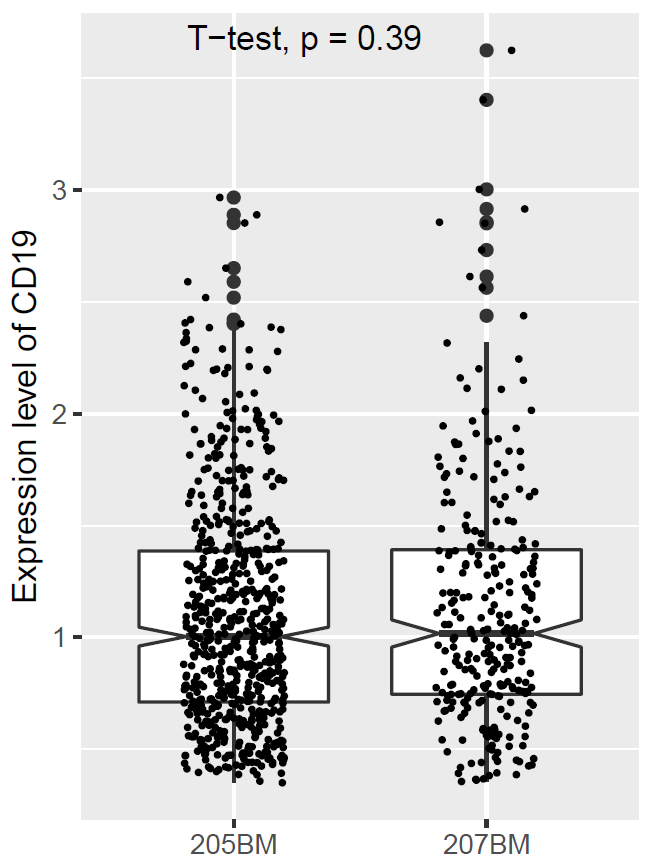


**Fig S14.** Violin plot of relative CD19 expression in tumor cells from samples 205BM and 207BM based on scRNA-Seq data.


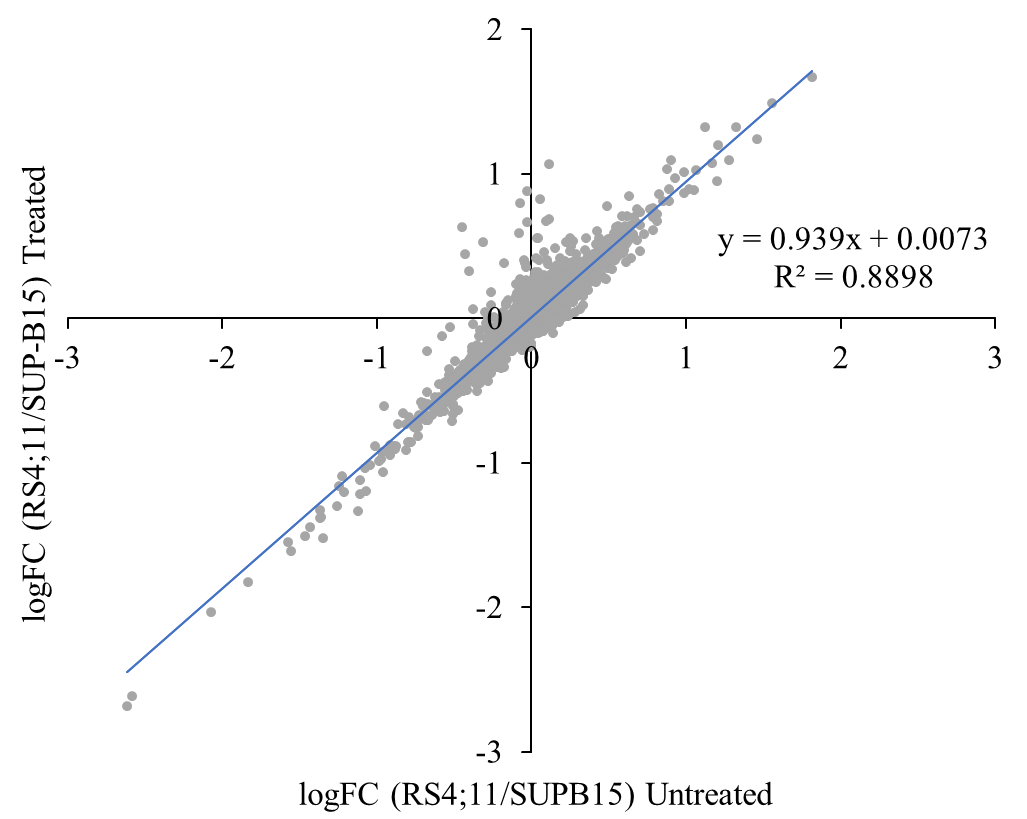


**Fig. S15.** The whole-genome gene expression comparison between RS4;11 and SUP-B15 cell line before and after Blinatumomab.

**Supplemental Tables**

**Table S1.** Sample information

| **Sample type** | **Sample Name** | **Time** | **Blinatumomab** | **Target cells** | **Estimated cells** |
| --- | --- | --- | --- | --- | --- |
| Cell line model | RU-16 | 16 h | 0 ng/mL | RS4;11 | 7609 |
|  | RT-16 | 16 h | 0.1 ng/mL | RS4;11 | 7961 |
|  | SU-16 | 16 h | 0 ng/mL | SUP-B15 | 7329 |
|  | ST-16 | 16 h | 0.1 ng/mL | SUP-B15 | 8683 |
|  | RU-48 | 48 h | 0 ng/mL | RS4;11 | 9266 |
|  | RT-48 | 48 h | 0.1 ng/mL | RS4;11 | 7961 |
|  | SU-48 | 48 h | 0 ng/mL | SUP-B15 | 6887 |
|  | ST-48 | 48 h | 0.1 ng/mL | SUP-B15 | 8241 |
| Patient-derived model | 205BM-B0 | 16 h | 0 ng/mL | / | 2747 |
|  | 205BM-B50 | 16 h | 10 ng/mL | / | 3910 |
|  | 207BM-B0 | 16 h | 0 ng/mL | / | 1082 |
|  | 207BM-B50 | 16 h | 10 ng/mL | / | 1791 |
|  | 207L-B0 | 16 h | 0 ng/mL | / | 1391 |
|  | 207L-B50 | 16 h | 10 ng/mL | / | 2319 |
