# Supplementary material for: Blinatumomab-induced T cell activation at single cell transcriptome resolution
Source: BMC Genomics. 2021 Mar 1;22:145. doi: 10.1186/s12864-021-07435-2 (PMC7923532; doi:10.1186/s12864-021-07435-2)
Supplement: Supplementary file 3 — Additional file 3. Detailed description of cell clustering and definition in the two models. [file 12864_2021_7435_MOESM3_ESM.docx]

**Supplementary text**

***Unsupervised clustering of the T cells and analysis of the T cell subpopulations***

As shown in Fig. 2B, for CD8+ cells, cluster TC0-CD8+ Naive T highly expressed the naïve marker genes TCF7, SELL, LEF1, CCR7 [1]. Cluster TC1-CD8+ TEM, with medium expression of effector factors [2-5] and low expression level of naïve marker genes, was composed of effector memory T cells. Cluster TC2-CD8+ CTL was characterized by high expression levels of effector factors and transcriptional factors associated of effector/memory differentiation, including EOMES, HOPX, TBX21, ZNF683 and ZEB2 [6-9]. The fourth cluster, TC3-CD8+ Activated T, shared similar expression of effector factors and transcriptional factors with TC2-CD8+ CTL. Meanwhile, cluster TC3-CD8+ Activated T also highly expressed the genes commonly expressed on activated T cells, like TNFRSF4, TNFRSF9, TNFRSF18, CD69 and IL2RA [10-14] and TC3-CD8+ Activated T predominantly showed up after blinatumomab treatment (Fig.2C) which mean TC3-CD8+ Activated T was composed of blinatumomab-activated CD8+ T cells. The last CD8+ T cell cluster, TC4-MAIT was characterized by specific expression of SLC4A10, KLRB1 and CCR6, which were related to mucosal associated invariant T cells (MAIT) [15].

Similarly, for CD4+ T cells (Fig. 2B), two clusters TC5-CD4+ Naïve T, TC6-CD4+ Naïve T-STAT1 highly expressed the naïve marker genes TCF7, SELL, LEF1 and CCR7. Also, TC7-CD4+ TCM and TC8-CD4+ TCM-IFIT3 expressing lower levels of naïve marker genes than naïve cell clusters were composed of central memory cells. And, comparing to TC5-CD4+ Naïve T and TC7-CD4+ TCM relevantly, TC6-CD4+ Naïve T-STAT1 and TC8-CD4+ TCM-IFIT3 were enriched in blinatumomab treatment group (Fig. 2C). TC10-CD4+ Activated T showed high expression of genes always expressed on activated T cells and mainly appeared after blinatumomab treatment. So TC10-CD4+ Activated T were composed of blinatumomab-activated CD4+ T cells. TC12-Tregs were characterized with high expression of Treg marker genes, IL2RA, FOXP3 and IKZF2 [16]. Cluster TC13-Activated T was the mixture of CD4+ and CD8+ T cells. Cluster TC13-Activated T highly expressed genes usually upregulated after T cell activation and was enriched after blinatumomab treatment.

TC14-DNT were double negative T cells without the expression CD4 and CD8 (Additional file 1, Fig. S3A). And TC14-DNT highly expressed the transcription factor regulating CD8 expression, CREM, which confirmed the identity of DN T cells [17]. Cluster TC15-gamma/delta T highly expressed the gamma/delta chain of TCR, TRDC1 and TRDC. The last cluster TC16-NKT was composed of natural killer T (NKT) cells showed high expression of effector factors and FCGR3A which is associated with the activation of natural killer cells (Fig. 2B) [18-20].

***Characterization of blinatumomab-activated Tregs***

To have an intrinsic analysis of blinatumomab-activated Tregs, we performed unsupervised clustering to TC12-Tregs and identified 3 Tregs sub clusters (Additional file 1, Fig. S4B) with the signature genes (Additional file 1, Fig. S4C). IFN-Tregs was characterized by the high expression of IFN-responsive genes (IFIT3, IFI6, ISG15, STAT1, EPSTI1 and MX1) and the increase of the proportion after blinatumomab treatment (Additional file 1, Fig. S4D). Then, Activated-Tregs highly expressed the activation markers (TNFRSF4, TNFRSF9, TNFRSF18 and MIR155HG) and was enriched after blinatumomab treatment, indicating cluster Activated-Tregs was composed of blinatumomab-activated Tregs (Additional file 1, Fig. S4D). And, the proportion of Resting-Tregs decreased after blinatumomab-treatment (Additional file 1, Fig. S4D).

**Identification of Blinatumomab-activated T cell clusters in B-ALL patient samples**

To explore the response of T cells to blinatumomab in patient samples and to validate the T cell responses we analyzed in cell line model samples, we performed the single cell RNA sequencing analysis to PBMCs and BMMCs from B-ALL patients (Additional file 1, Table S1). In total, 13240 cells passed the data quality control and 5 main clusters were identified with the signature genes (Additional file 1, Fig. S7A). We defined clusters PC0, PC1, PC2, PC3 and PC4 to be composed of tumor cells from patient #207, tumor cells from patient #205, T cells, B cells and erythrocytes relevantly according to their expression of well-known marker genes of each cell type.

Then we performed the unsupervised clustering of a total of 2271 T cells in cluster PC2 and identified 9 T cell subclusters with its signature genes (Fig. 4A, Additional file 5, Table S4). According to the expression distribution of CD4, CD8A and CD8B (Additional file 1, Fig. S7C), 3 CD8+ T cell clusters (PTC0-CD8+ TEM, PTC1-CD8+ CTL, PTC2-CD8+ Activated T), 2 CD4+ T cell clusters (PTC7-CD4+ TH-NFKB2, PTC8-CD4+ TH-BIRC3) and 4 mixed T cell clusters (PTC3-Naive T, PTC4-TCM, PTC5-IFN-T, PTC6-Activated T) were identified. Moreover, the expression of known functional markers in each cluster indicated the subtypes of all the T cell clusters (Fig. 4B). Also, the number of shared signature genes of clusters from cell line model samples and patient samples were calculated to compare the similarities of the relevant clusters from these two samples (Additional file 1, Fig. S7D). Cluster PTC0-CD8+ TEM was characterized for the lower expression levels of cytotoxic factors and selected transcriptional factors, compared to cluster PTC1-CD8+ CTL which was composed of cytotoxic CD8+ T cells. Cluster PTC2-CD8+ Activated T was composed of the blinatumomab-activated CD8+ T cells for its high expression of activation markers. Also, top 20 signature genes of PTC0-CD8+ TEM, PTC1-CD8+ CTL and PTC2-CD8+ Activated T shared the highest similarity with that of TC1-CD8+ TEM, TC2-CD8+ CTL and TC3-CD8+ Activated T relevantly. Cluster PTC3-Naive T highly expressed the naïve markers genes. Cluster PTC4-T-S100A11 was mainly composed of effector T cells for the similarity of top 20 signature genes of PTC4-T-S100A11 to that of TC9-CD4+ TH-KLRB1. Cluster PTC5-IFN-T was characterized by the high expression of IFN-responsive genes and shared 9 signature genes with cluster TC6-CD4+ Naïve T-STAT1. Cluster PTC6-Activated T was characterized by its high expression levels of activation markers and shared 10 signature genes with cluster TC13-Activated T. These results showed the T cell types we identified in patient samples were comparable with these identified in cell line samples.

Then, we combined the untreated groups (205BM-B0, 207BM-B0, 207L-B0) and combined treated groups (205BM-B50, 207BM-B50, 207L-B50) to compare the T cell sub cluster proportions before and after blinatumomab treatment and found similar changes in patient samples compared with those in cell line model samples. The proportion of PTC2-CD8+ activated T, PTC5-IFN-T, PTC6-Activated T increased after blinatumomab treatment (Additional file 1, Fig. S7E). These data indicated similar T cell state transition found in patient samples compared with that in cell line model samples, including the activation from CD8+ T_EM_ cells and naïve cells, as well as the IFN-responsive state transition.

Above all, the similar results obtained in patient samples confirmed the analysis in cell line model samples and suggested the gene expression changes we identified were able to reveal the T cell responses during blinatumomab-mediated cytotoxicity.

**Reference**

1. Förster R, Davalosmisslitz AC, Rot A: **CCR7 and its ligands: balancing immunity and tolerance**. *Nature Reviews Immunology* 2008, **8**(5):362.

2. Trapani JA, Smyth MJ: **Functional significance of the perforin/granzyme cell death pathway**. *Nature Reviews Immunology* 2002, **2**(10):735-747.

3. Yamashita Y, Al E: **Perforin and granzyme expression in cytotoxic T-cell lymphomas**. *Mod Pathol* 1998, **11**(4):313-323.

4. Krensky AM, Clayberger C: **Biology and clinical relevance of granulysin**. *Tissue Antigens* 2010, **73**(3):193-198.

5. Medley QG, Kedersha N, ., O'Brien S, ., Tian Q, ., Schlossman SF, Streuli M, ., Anderson P, . **Characterization of GMP-17, a granule membrane protein that moves to the plasma membrane of natural killer cells following target cell recognition**. *Proc Natl Acad Sci U S A* 1996, **93**(2):685-689.

6. Dominguez CX, Amezquita RA, Guan T, Marshall HD, Joshi NS, Kleinstein SH, Kaech SM: **The transcription factors ZEB2 and T-bet cooperate to program cytotoxic T cell terminal differentiation in response to LCMV viral infection**. *Journal of Experimental Medicine* 2015, **212**(12):2041-2056.

7. Mackay L, Minnich M, Kragten N, Liao Y, Nota B, Seillet C, Zaid A, Man K, Preston S, Freestone D *et al*: **Hobit and Blimp1 instruct a universal transcriptional program of tissue residency in lymphocytes**, vol. 352; 2016.

8. Pearce EL, Mullen AC, Martins GA, Krawczyk CM, Hutchins AS, Zediak VP, Monica B, Dicioccio CB, Gross DA, Chai-An M: **Control of effector CD8+ T cell function by the transcription factor Eomesodermin**. *Science* 2003, **302**(5647):1041-1043.

9. Mariotto A, Pavlova O, Park HS, Huber M, Hohl D: **HOPX: The Unusual Homeodomain-Containing Protein**. *Journal of Investigative Dermatology* 2016, **136**(5):905-911.

10. Ziegler SF, Ramsdell F, Alderson MR: **The activation antigen CD69**. *STEM CELLS* 1994, **12**(5):456-465.

11. Liao W, Lin J-X, Leonard WJ: **Interleukin-2 at the crossroads of effector responses, tolerance, and immunotherapy**. *Immunity* 2013, **38**(1):13-25.

12. Nocentini G, Riccardi C: **GITR: A Modulator of Immune Response and Inflammation**. *Oxygen Transport to Tissue XXXIII* 2009, **647**:156-173.

13. Redmond WL, Ruby CEWeinberg AD: **The role of OX40-mediated co-stimulation in T-cell activation and survival**. *Critical Reviews in Immunology* 2009, **29**(3):187-201.

14. Chao W, Lin GHY, Mcpherson AJ, Watts TH: **Immune regulation by 4-1BB and 4-1BBL: complexities and challenges**. *Immunological Reviews* 2010, **229**(1):192-215.

15. Kurioka A, Walker LJ, Klenerman P, Willberg CB: **MAIT cells: new guardians of the liver**. *Clinical & Translational Immunology* 2016, **5**(8):e98.

16. Bhairavabhotla R, Kim YC, Glass DD, Escobar TM, Patel MC, Zahr R, Nguyen CK, Kilaru GK, Muljo SA, Shevach EM: **Transcriptome profiling of human FoxP3+ regulatory T cells**. *Human Immunology* 2016, **77**(2):201-213.

17. Hedrich CM, Rauen T, Crispin JC, Koga T, Ioannidis C, Zajdel M, Kyttaris VC, Tsokos GC: **cAMP-responsive Element Modulator α (CREMα) trans-Represses the Transmembrane Glycoprotein CD8 and Contributes to the Generation of CD3+CD4−CD8− T Cells in Health and Disease**. *Journal of Biological Chemistry* 2013, **288**(44):31880-31887.

18. L Gansert J, Kiessler V, Engele M, Wittke F, Röllinghoff M, Krensky A, Porcelli S, L Modlin R, Stenger S: **Human NKT Cells Express Granulysin and Exhibit Antimycobacterial Activity**, vol. 170; 2003.

19. Ortaldo JR, Winkler-Pickett RT, Yagita H, Young HA: **Comparative studies of CD3 − and CD3 + CD56 + cells: Examination of morphology, functions, T cell receptor rearrangement, and pore-forming protein expression**. *Cellular Immunology* 1991, **136**(2):486.

20. Kronenberg M: **Toward an understanding of NKT cell biology: progress and paradoxes**. *Annual Review of Immunology* 2005, **23**(1):877-900.
